# High glucose enhances the activation of NLRP3 inflammasome by ambient fine particulate matter in alveolar macrophages

**DOI:** 10.1186/s12989-023-00552-8

**Published:** 2023-11-02

**Authors:** Yiqun Mo, Luke Mo, Yue Zhang, Yuanbao Zhang, Jiali Yuan, Qunwei Zhang

**Affiliations:** 1 Department of Epidemiology and Population Health, School of Public Health and Information Sciences, University of Louisville, 485 E. Gray Street, Louisville, KY 40202 USA; 2 Northwestern University Feinberg School of Medicine, Chicago, IL 60611 USA

**Keywords:** Ambient fine particulate matter (PM_2.5_), Alveolar macrophage, High glucose, NLRP3 inflammasome, IL-1β, MMP-9

## Abstract

**Background:**

Epidemiological studies have demonstrated that individuals with preexisting conditions, including diabetes mellitus (DM), are more susceptible to air pollution. However, the underlying mechanisms remain unclear. In this study, we proposed that a high glucose setting enhances ambient fine particulate matter (PM_2.5_)-induced macrophage activation and secretion of the proinflammatory cytokine, IL-1β, through activation of the NLRP3 inflammasome, altering the balance between matrix metalloproteinases (MMPs) and tissue inhibitors of MMPs (TIMPs).

**Results:**

Exposure of mouse alveolar macrophages to non-cytotoxic doses of PM_2.5_ led to upregulation of IL-1β, activation of the NLRP3 inflammasome, increased nuclear translocation of the transcription factor NF-κB, increased generation of reactive oxygen species (ROS), and increased expression and enzymatic activity of MMP-9; these effects were enhanced when cells were pretreated with high glucose. However, pretreatment in a high glucose setting alone did not induce significant changes. ROS generation following PM_2.5_ exposure was abolished when cells were pretreated with ROS scavengers such as Trolox and superoxide dismutase (SOD), or with an NADPH oxidase inhibitor, DPI. Pretreatment of cells with DPI attenuated the effects of a high glucose setting on PM_2.5_-induced upregulation of IL-1β, activation of the NLRP3 inflammasome, and nuclear translocation of NF-κB. In addition, enhancement of PM_2.5_-induced expression and enzymatic activity of MMP-9 following high glucose pretreatment was not observed in primary alveolar macrophages obtained from NLRP3 or IL-1R1 knockout (KO) mice, where pro-IL-1β cannot be cleaved to IL-1β or cells are insensitive to IL-1β, respectively.

**Conclusions:**

This study demonstrated that exposure of mouse alveolar macrophages to PM_2.5_ in a high glucose setting enhanced PM_2.5_-induced production of IL-1β through activation of the NLRP3 inflammasome and nuclear translocation of NF-κB due to PM_2.5_-induced oxidative stress, leading to MMP-9 upregulation. The key role of NADPH oxidase in PM_2.5_-induced ROS generation and activation of the IL-1β secretion pathway and the importance of IL-1β secretion and signaling in PM_2.5_-induced increases in MMP-9 enzymatic activity were also demonstrated. This study provides a further understanding of the potential mechanisms underlying the susceptibility of individuals with DM to air pollution and suggests potential therapeutic targets.

**Supplementary Information:**

The online version contains supplementary material available at 10.1186/s12989-023-00552-8.

## Background

Air pollution, composed of particulate matter (PM) and gases such as sulfur and nitrogen dioxide, is a serious public health issue. Fine ambient PM of diameter less than or equal to 2.5 µm (PM_2.5_) can penetrate from the mouth and throat deep into the lungs, posing serious health problems [1]. Epidemiological studies suggest a link between particulate air pollution and increased morbidity and mortality from respiratory and cardiovascular diseases [2–5]. In addition, epidemiological studies and animal experiments have shown that individuals with preexisting conditions, such as diabetes mellitus, chronic obstructive pulmonary disease (COPD), asthma, fatty liver disease, sepsis, etc., are more susceptible to air pollution [6–10]. However, the mechanisms underlying this susceptibility are still not clarified.

Diabetes mellitus (DM) is the most common endocrine disorder in humans. DM is principally characterized by hyperglycemia, resulting in microvascular and macrovascular complications, such as diabetic retinopathy, coronary artery disease, etc., that increase mortality and reduce the quality of life. Diabetes can increase the severity and clinical course of several pulmonary diseases including asthma, COPD, fibrosis, pulmonary hypertension, lung cancer, etc., which is mainly associated with the pro-inflammatory and proliferative properties of diabetes [11]. Several epidemiological studies have shown that people with DM are especially sensitive to the effects of PM on daily mortality and pulmonary and cardiovascular disease hospitalizations [9]. However, a biological mechanism linking PM exposure to exacerbated impairment of pulmonary and cardiovascular function in people with DM is still unknown.

The first step of PM-induced pulmonary injury is the inflammatory response, where immune cells such as macrophages, neutrophils, etc. are excessively activated. A variety of cytokines have been implicated in the pathogenesis of PM-induced lung inflammation and injury [12]. IL-1β is one of a family of pro-inflammatory cytokines thought to be involved in the initiation of the inflammatory process, contributing to acute and chronic inflammation [13, 14]. Excessive pro-inflammatory activity driven by IL-1β is a major pathophysiological event in several autoimmune, neurodegenerative, and metabolic diseases. IL-1β is released by a highly regulated process, in which caspase-1-mediated cleavage of pro-IL-β is the rate-limiting step [14]. Previous studies have shown that exposure to either PM or high glucose causes IL-1β secretion in macrophages or monocytes [15–17]. Though the detailed mechanisms by which IL-1β is induced are unknown, previous studies have shown that activation of the NLRP3 inflammasome and/or toll-like receptors (TLRs), oxidative stress, nuclear translocation of transcription factor NF-κB, etc., may be involved in PM or high glucose-induced IL-1β secretion [15–17].

Although several studies have addressed the modulation of the macrophage response to PM, few studies have focused on the combined effects of PM and high glucose on macrophages, especially concerning the production of IL-1β and the underlying mechanisms responsible. Hence, the purpose of this study was to evaluate the effects of PM_2.5_ on mouse alveolar macrophages with and without high glucose pretreatment and to identify the potential mechanisms involved in the enhanced susceptibility of macrophages to PM_2.5_ in a high glucose environment. The hypothesis was that exposure of alveolar macrophages to PM_2.5_ in the setting of high glucose would result in enhanced activation of alveolar macrophages, leading to increased production of IL-1β through activation of NLRP3 inflammasome and/or TLRs and increased nuclear translocation of transcription factor NF-κB by PM_2.5_-induced oxidative stress, finally resulting in MMPs/TIMPs imbalance.

## Methods

### Fine ambient particulate matter (PM_2.5_)

Fine ambient particulate matter (PM_2.5_), a Standard Reference Material® (SRM 2786) with a mean diameter of approximately 2.8 μm, was purchased from the National Institute of Standards and Technology (NIST) (Gaithersburg, MD, USA). Its certificate of analysis is available online (https://tsapps.nist.gov/srmext/certificates/2786.pdf). In this study, PM_2.5_ was prepared at a concentration of 5 mg/mL in physiological saline and diluted with physiological saline if necessary. The PM_2.5_ was vortexed thoroughly before each experiment.

### Chemicals and reagents

Primary antibodies including anti-IL-1β (D6D6T) (cat. no. 31202, 1:500), anti-cleaved IL-1β (E7V2A) (cat. no. 63124, 1:1000), anti-NLRP3 (D4D8T) (cat. no. 15101, 1:1000), anti-TLR2 (E1J2W) (cat. no. 13744, 1:1000), anti-Histone H3 (cat. no. 9715, 1:2000), and anti-β-actin (E4D9Z) (cat. no. 58169, 1:2000) antibodies, and secondary antibodies including HRP-conjugated goat anti-rabbit IgG (cat. no. 7074, 1:2000) and horse anti-mouse IgG (cat. no. 7076, 1:2000) were purchased from the Cell Signaling Technology (Beverly, MA, USA). Anti-NF-κB p65 (F-6) (cat. no. sc-8008, 1:200) was obtained from Santa Cruz Biotechnology (Santa Cruz, CA, USA).

### Cell culture and PM_2.5_ and/or high glucose treatment

Immortalized mouse alveolar macrophages MH-S were purchased from American Type Culture Collection (ATCC, cat. no. CRL-2019, Manassas, VA, USA) and cultured in an incubator with a humidified atmosphere of 5% CO_2_ at 37 °C. The complete cell culture medium contained RPMI 1640 medium (cat. no. 10–043-CV, without glucose, Corning, Manassas, VA, USA) supplemented with 10% FBS, 100 IU/mL penicillin, 100 μg/mL streptomycin (Corning), 0.05 mM 2-mercaptoethanol (Gibco, Grand Island, NY, USA), and 5 mM glucose (Acros Organics, NJ, USA).

To determine the mRNA or protein expression levels of pro-inflammatory cytokines (IL-1β and IL-18), components of NLRP3 inflammasome (NLRP3 and caspase-1), Toll-like receptors (TLR2 and TLR4), NF-κB p65 (nuclear and cytoplasmic), MMPs (MMP-2 and MMP-9), and TIMPs (TIMP-1 and TIMP-2) in MH-S macrophages after PM_2.5_ exposure, the cells were treated with 25 or 50 μg/mL of PM_2.5_ for 1, 3, 6, 12, or 24 h (time-response study), or with 25, 50, and 100 μg/mL of PM_2.5_ for 6 h (dose–response study). To determine whether the effects of PM_2.5_ were enhanced at a high glucose setting, the MH-S cells were cultured in complete medium containing 30 mM glucose. The control cells were cultured in complete medium only. Mannitol was used as an osmolality control. 3 × 10^6^ cells in 20 mL medium were seeded in each 75 cm^2^ flask (day 0). The medium was refreshed on day 4 and day 7. On day 9, the cells were split, and 3 × 10^6^ cells were seeded for another cycle. After 2 cycles (18 days), the cells were collected and 1 × 10^6^ cells in 2 mL medium were seeded in each well of 6-well plates. After overnight culture, the medium was refreshed, and the cells were treated with 25 or 50 μg/mL of PM_2.5_ (20 μL of 2.5 or 5 mg/mL of PM_2.5_) for 3 h (for IL-1β, NLRP3, or TLR2 mRNA), 6 h (for IL-1β, NLRP3, or TLR2 protein by Western blot), or 24 h (for MMPs and TIMPs). The cells treated with physiological saline (20 μL) were used as a control.

### Cytotoxicity of PM_2.5_ and/or glucose on mouse alveolar macrophages MH-S

The cells were seeded in 96-well plates. After overnight culture, the cells were treated with 0, 25, 50, 100, 200, and 400 μg/mL of PM_2.5_ or 5, 10, 20, 30, 40, and 50 mM of glucose for 24 h. The cytotoxicity of PM_2.5_ or glucose in MH-S cells was determined by two different methods: CellTiter 96 AQ_ueous_ Non-Radioactive Cell Proliferation Assay (MTS assay) (Promega, Madison, WI, USA) and alamarBlue™ assay (Invitrogen, Eugene, OR, USA) according to the manufacturer’s instructions. To observe whether there was cytotoxicity with PM_2.5_ treatment in a high glucose environment, the cells were pretreated with 30 mM glucose for 18 days followed by 25 or 50 μg/mL of PM_2.5_ treatment for another 24 h. Mannitol was used as an osmolality control.

### Total RNA isolation and RT-qPCR

Total RNA was isolated from the cells by using TRIzol® reagent (Sigma-Aldrich, St. Louis, MO, USA), and RT-qPCR was performed as described previously [18, 19]. 2 µg total RNA was reverse-transcribed into cDNA by using M-MLV reverse transcriptase (Promega, Madison, WI, USA). qPCR was performed by using iTaq™ Universal SYBR® Green Supermix (Bio-Rad, Hercules, CA, USA) on a BioRad iQ5 Multicolor Real-Time PCR Detection System (Bio-Rad). The PCR reaction was performed as follows: 40 cycles at 94 °C for 10 s, at 58 °C for 45 s, and at 72 °C for 45 s. Data were quantified by using the 2^−ΔΔCt^ (Livak) method [20]. β-actin was used as an internal control. All the primers used were listed in Table 1.

**Table 1:** Primers used for RT-qPCR

Gene	Forward (5’ → 3’)	Reverse (5’ → 3’)	Product size
IL-1β	TCATGGGATGATGATGATAACCTGCT	CCCATACTTTAGGAAGACACGGATT	503 bp
IL-18	GACTCTTGCGTCAACTTCAAGG	CAGGCTGTCTTTTGTCAACGA	169 bp
NLRP3	GCAGGAGGAAGACTTTGTGC	AGGAGATGTCGAAGCAGCAT	461 bp
Caspase-1	TACCTGGCAGGAATTCTGGA	ATGATCACCTTGGGCTTGTC	596 bp
TLR2	ACAGCTACCTGTGTGACTCTCCGCC	GGTCTTGGTGTTCATTATCTTGCGC	602 bp
TLR4	GCTTTCACCTCTGCCTTCAC	AGGCGATACAATTCCACCTG	259 bp
MMP-2	CCAACTACGATGATGAC	ACCAGTGTCAGTATCAG	233 bp
MMP-9	ACCACCACAACTGAACCACA	ACCAACCGTCCTTGAAGAAA	304 bp
TIMP-1	ACCACCTTATACCAGCGTTA	AAACAGGGAAACACTGTGCA	305 bp
TIMP-2	CACCCGCAACAGGCGTTTTG	ATCTTGCCATCTCCTTCTGC	269 bp
β-actin	GGCATTGTTACCAACTGGGAC	ACCAGAGGCATACAGGGACAG	219 bp

### Protein extraction and Western blot

Total protein was isolated from the cells by RIPA lysis buffer (Santa Cruz Biotechnology, Santa Cruz, CA, USA) while nuclear and cytoplasmic proteins were extracted by using NE-PER™ Nuclear and Cytoplasmic Extraction Reagent (Thermo Scientific, Rockford, IL, USA) according to the manufacturer’s instructions. Western blot was performed as described previously [18, 21]. The expression of β-actin was used as an internal reference for cytoplasmic or total protein, and histone H3 was for nuclear protein. Immunoreactive bands were quantified by using NIH ImageJ software (http://imagej.nih.gov/ij/). Uncropped versions of Western blots were shown in Additional file 5.

### Measurement of ROS

ROS generation in mouse alveolar macrophages MH-S after PM_2.5_ and/or high glucose exposure was determined by using 2', 7'-dichlorodihydrofluorescein diacetate (H_2_DCF-DA, Molecular Probes, Eugene, OR, USA). The cells seeded in 96-well plates were pretreated with 5 µM of H_2_DCF-DA for 2 h before the cells were exposed to 0, 6.3, 12.5, 25, and 50 μg/mL of PM_2.5_ for 12 h, or to 50 μg/mL of PM_2.5_ for 3, 6, 12, and 24 h. To determine the effects of PM_2.5_ on ROS generation in MH-S cells with high glucose pretreatment, the cells were pretreated with 30 mM of glucose for 18 days. Then the cells were seeded and pretreated with 5 µM of H_2_DCF-DA for 2 h, followed by treatment with 50 μg/mL of PM_2.5_ for another 12 h. The cells treated with physiological saline were used as the control, and mannitol was used as an osmolality control. The DCF fluorescence was measured by using a Synergy HT microreader (BioTek, Winooski, VT, USA) at λex485/ λem528.

To observe the effects of ROS scavenges or inhibitors on ROS generation in MH-S cells after PM_2.5_ exposure, the following reagents were used: (1) 100 μM of Trolox (Sigma-Aldrich, St. Louis, MO, USA), a water-soluble analog of vitamin E which has an antioxidant effect; (2) 10 μM of diphenyleneiodonium chloride (DPI) (Alexis, San Diego, CA, USA), a specific inhibitor of NADPH oxidase; and (3) 300 U/mL of superoxide dismutase (SOD) (Sigma-Aldrich), an enzyme that catalyzes the dismutation of the superoxide radical into oxygen and hydrogen peroxide. The cells were pre-treated with ROS scavenges or inhibitors for 2 h and 5 μM of H2-DCFDA for another 2 h, followed by 25 or 50 μg/mL of PM_2.5_ treatment for 12 h. The fluorescence values were measured as described above.

MitoSOX™ Red Mitochondrial Superoxide Indicator (Invitrogen, Eugene, OR, USA) was used to detect mitochondrial ROS generation in MHS cells after PM_2.5_ exposure according to the manufacturer’s instructions. Briefly, the cells were pretreated with 5 µM of MitoSOX™ for one hour before 0, 6.3, 12.5, 25, and 50 μg/mL of PM_2.5_ exposure for another 12 h. The fluorescence at λex530/ λem590 was recorded by a Synergy HT microreader (BioTek, Winooski, VT, USA).

### Measurement of cytokines and MMP-2/9 by ELISA

The levels of IL-1β in the cell culture media were determined by Mouse IL-1beta ELISA kit (cat. no. BMS6002, Invitrogen by Thermo Fisher Scientific, Vienna, Austria) while the MMP-2 and MMP-9 protein levels were analyzed by Mouse MMP-2 or MMP-9 PicoKine™ ELISA Kit (cat. no. EK0460 or EK0466, Boster Biological Technology, Pleasanton, CA, USA) according to the manufacturer’s instructions.

### Gelatin zymography assay

The enzymatic activities of MMP-2 and MMP-9 were determined by gelatin zymography assay as described in our previous studies [19, 22]. Briefly, the cells were cultured in FBS-free media for 24 h prior to PM_2.5_ exposure. At the end of exposure, the cell culture media were collected, and an equal volume of media was loaded in each lane of 10% SDS-PAGE copolymerized with 0.5 mg/mL gelatin, which was used as the substrate under nonreducing conditions. After washing 4 times (15 min each) with renaturing buffer [50 mM Tris–HCl buffer (pH 7.5), 2.5% Triton X-100] at room temperature, the gels were incubated in calcium assay buffer [50 mM Tris–HCl buffer (pH 7.5), 0.2 M NaCl, 7.55 mM CaCl_2_, 1 μM ZnCl_2_, and 1% Triton X-100] at 37 °C overnight. After briefly washing with ddH_2_O, the gel was stained with 0.1% Coomassie Brilliant Blue R-250 (Bio-Rad, Hercules, CA, USA) at room temperature for one hour, then destained with 10% acetic acid until the clear bands were observed.

### Isolation of alveolar macrophages from mice

Animal use was reviewed and approved by the University of Louisville Institutional Animal Care and Use Committee. Wild-type C57BL/6J (JAX stock no. 000664), NLRP3 knockout (KO) (B6.129S6-*Nlrp3*^*tm1Bhk*^/J, JAX stock no. 021302), and IL-1R1 KO (B6.129S7-*Il1r1*^*tm1Imx*^/J, JAX stock no. 003245) mice were purchased from The Jackson Laboratory (Bar Harbor, ME, USA), and both strains of homozygous KO mice are viable, fertile, and bred in our university animal facility. The mice were housed in an air-conditioned room (temperature of 20 ± 2 °C, relative humidity of 60 ± 10%) with a 12 h light and 12 h dark cycle environment and with free access to food and water.

Primary alveolar macrophages were isolated from C57BL/6J, NLRP3 KO, and IL-1R1 KO mice by bronchoalveolar lavage (BAL) as described previously [23, 24]. 0.8 mL of ice-cold FBS- and glucose-free RPMI 1640 medium supplemented with 100 IU/mL penicillin, 100 μg/mL streptomycin, and 0.4 mM EDTA was used to lavage the lungs. Each mouse was lavaged six times, and approximately 1 × 10^5^ macrophages were obtained from each mouse. The lavage fluid from the same strain of mice was combined and centrifugated at 200 × *g* and 4 °C for 10 min. After centrifugation, the cells were resuspended at 2 × 10^5^ or 6 × 10^5^ cells/mL in RPMI 1640 medium supplemented with 10% FBS, 100 IU/mL penicillin, 100 μg/mL streptomycin, 0.05 mM 2-mercaptoethanol, and 5 mM glucose. Then, 1 × 10^5^ (for IL-1β and NLRP3 mRNA) or 3 × 10^5^ (for MMP-2/9 protein and activity) cells were seeded into each well of a 24-well plate. The cells were treated with 50 μg/mL of PM_2.5_ for 3 h (for IL-1β and NLRP3 mRNA) or 24 h (for MMP-2/9 protein and activity) with/without 30 mM glucose pretreatment for 24 h. After treatment, the cells or the cell culture media were collected. If the cell culture media would be used for the determination of MMP-2 and MMP-9 protein levels by ELISA or their activities by gelatin zymography assay, FBS-free medium was used to culture the cells. The above procedures were repeated three times.

### Statistical analysis

Data were expressed as the mean ± SEM, and the differences were analyzed by one-way analysis of variance (ANOVA) followed by Dunnett (for comparison with the control) or Bonferroni (for all pairwise comparisons) post-hoc test or two-way ANOVA followed by the Holm-Sidak test by using SigmaPlot 13.0 software (Systat Software, Inc., San Jose, CA, USA). A difference was considered statistically significant when a *p*-value was less than 0.05.

## Results

### Cytotoxicity of PM_2.5_ and/or glucose on mouse alveolar macrophages MH-S

**Fig. 1:**
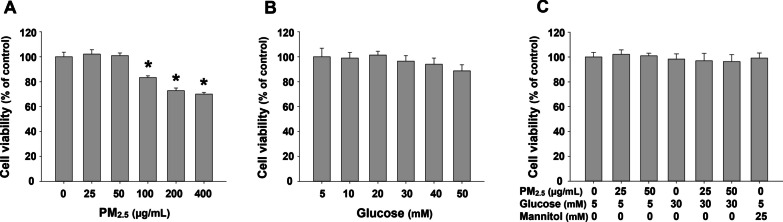
Cytotoxicity of PM_2.5_ and/or glucose on MH-S cells. **a** and **b** The cells were treated with PM_2.5_ or glucose for 24 h. **c** The cells were pretreated with 30 mM of glucose for 18 days, followed by treatment with PM_2.5_ for 24 h. Mannitol was used as an osmolality control. The cells treated with physiological saline were used as control. Cytotoxicity was determined by MTS assay (Promega). Data are shown as mean ± SEM (*n* = 6). * *p* < 0.05 vs. control

To find the appropriate non-cytotoxic doses for the following mechanism study, the cytotoxicity of PM_2.5_ or glucose in mouse alveolar macrophages MH-S was at first determined. The results showed that exposure of MH-S cells to 50 μg/mL or less of PM_2.5_ for 24 h did not cause significant cytotoxicity although exposure to 100 μg/mL or above of PM_2.5_ did by using MTS assay (Fig. 1a), which was further confirmed by using alamarBlue™ assay (Additional file 1). On the other hand, treatment with as high as 50 mM glucose did not result in any cytotoxicity in MH-S cells (Fig. 1b). Therefore, in the subsequent experiments, non-cytotoxic doses of PM_2.5_ (≤ 50 µg/mL) and glucose (30 mM) were chosen to observe the effects of PM_2.5_ with/without high glucose pretreatment on mouse alveolar macrophages. When the cells were pretreated with 30 mM glucose or mannitol (as an osmolality control) for 18 days followed by 25 or 50 µg/mL of PM_2.5_ treatment for another 24 h, no cytotoxicity was observed (Fig. 1c).

### Exposure to PM_2.5_ caused upregulation of IL-1β, NLRP3, and TLR2 in MH-S cells

**Fig. 2:**
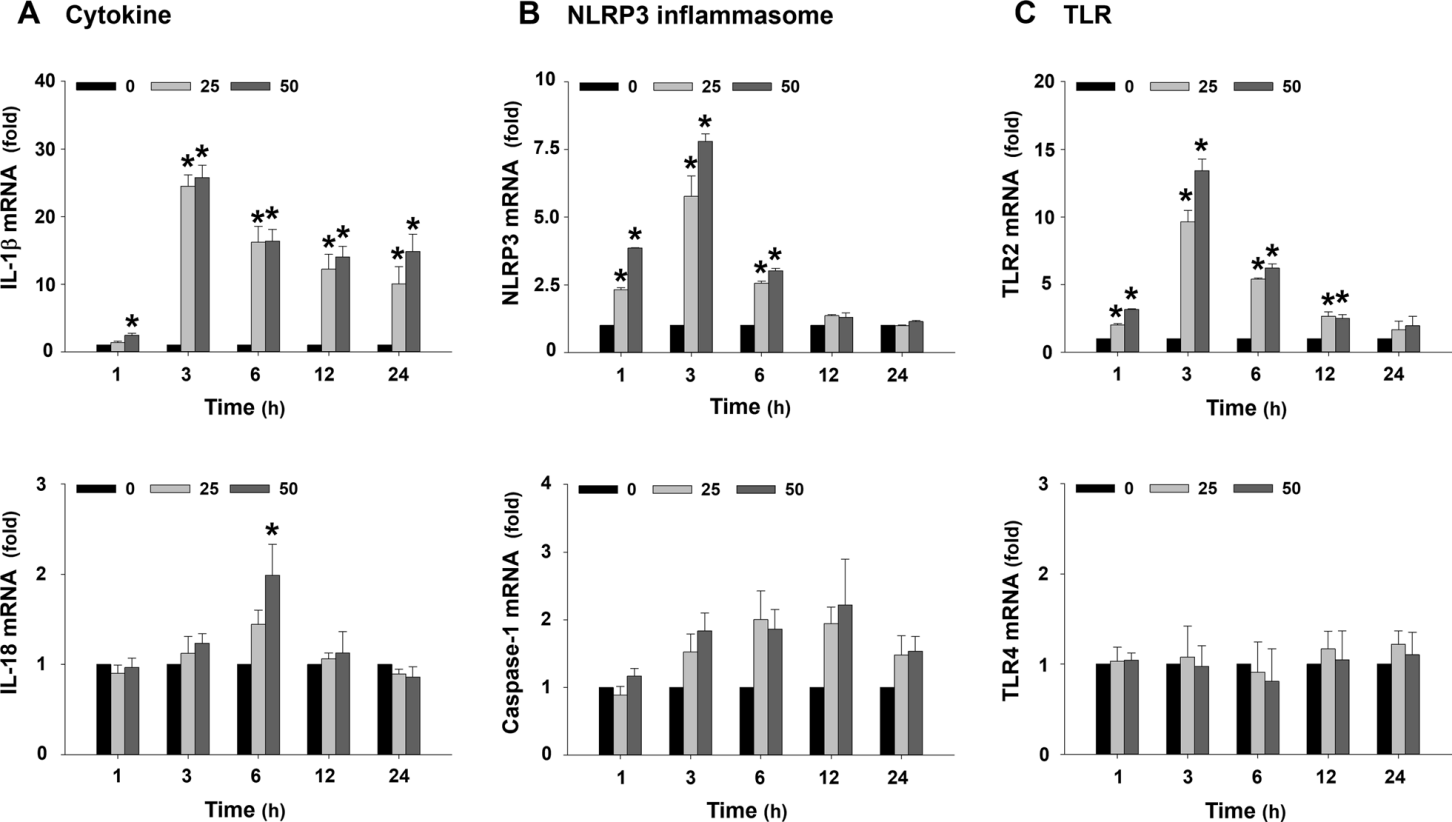
mRNA expression levels of pro-inflammatory cytokines (**a**), components of NLRP3 inflammasome (**b**), and TLRs (**c**) in MH-S cells exposed to PM_2.5_ (dose- and time-response studies). The cells were treated with 25 or 50 μg/mL of PM_2.5_ for 1, 3, 6, 12, and 24 h. The cells treated with physiological saline were used as control. The mRNA expression of the gene was determined by RT-qPCR and normalized to the β-actin expression in the same sample. Data are shown as mean ± SEM (*n* = 3). * *p* < 0.05 vs. control

The expression levels of pro-inflammatory cytokines (IL-1β and IL-18), components of NLRP3 inflammasome (NLRP3 and caspase-1), and TLRs (TLR2 and TLR4) in mouse alveolar macrophages MH-S were determined at both mRNA level by RT-qPCR and protein level by Western blot. For RT-qPCR, the cells were exposed to 25 or 50 μg/mL of PM_2.5_ for 1, 3, 6, 12, and 24 h. The results showed that 50 μg/mL of PM_2.5_ exposure caused significantly increased IL-1β mRNA expression as early as 1 h after exposure. Exposure of the cells to both 25 and 50 μg/mL of PM_2.5_ for 3 h caused near 25-fold increase in IL-1β mRNA, which maintained an increase at 6, 12, and 24 h after PM_2.5_ exposure (Fig. 2a). However, only 50 μg/mL of PM_2.5_ exposure for 6 h caused a twofold increase in IL-18 mRNA, which returned to baseline level at 12 and 24 h after PM_2.5_ exposure (Fig. 2a). In addition, 25 and 50 μg/mL of PM_2.5_ exposure also caused significant upregulation of NLRP3 and TLR2, but not caspase-1 and TLR4, at the similar pattern as IL-1β mRNA expression (Fig. 2b, c).

**Fig. 3:**
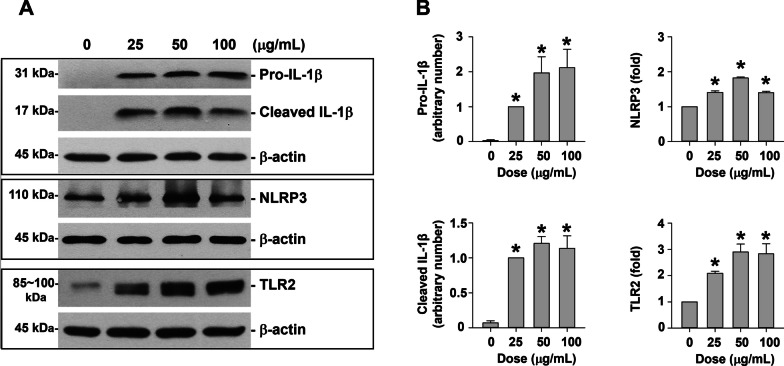
Dose–response study of protein expression of IL-1β, NLRP3, and TLR2 in MH-S cells exposed to PM_2.5_. The cells were treated with 25, 50, and 100 μg/mL of PM_2.5_ for 6 h. The cells treated with physiological saline were used as control. **a** is the Western blot result of a single experiment while **b** is the normalized results of Western blots. Data are shown as mean ± SEM (*n* = 3). * *p* < 0.05 vs. control

**Fig. 4:**
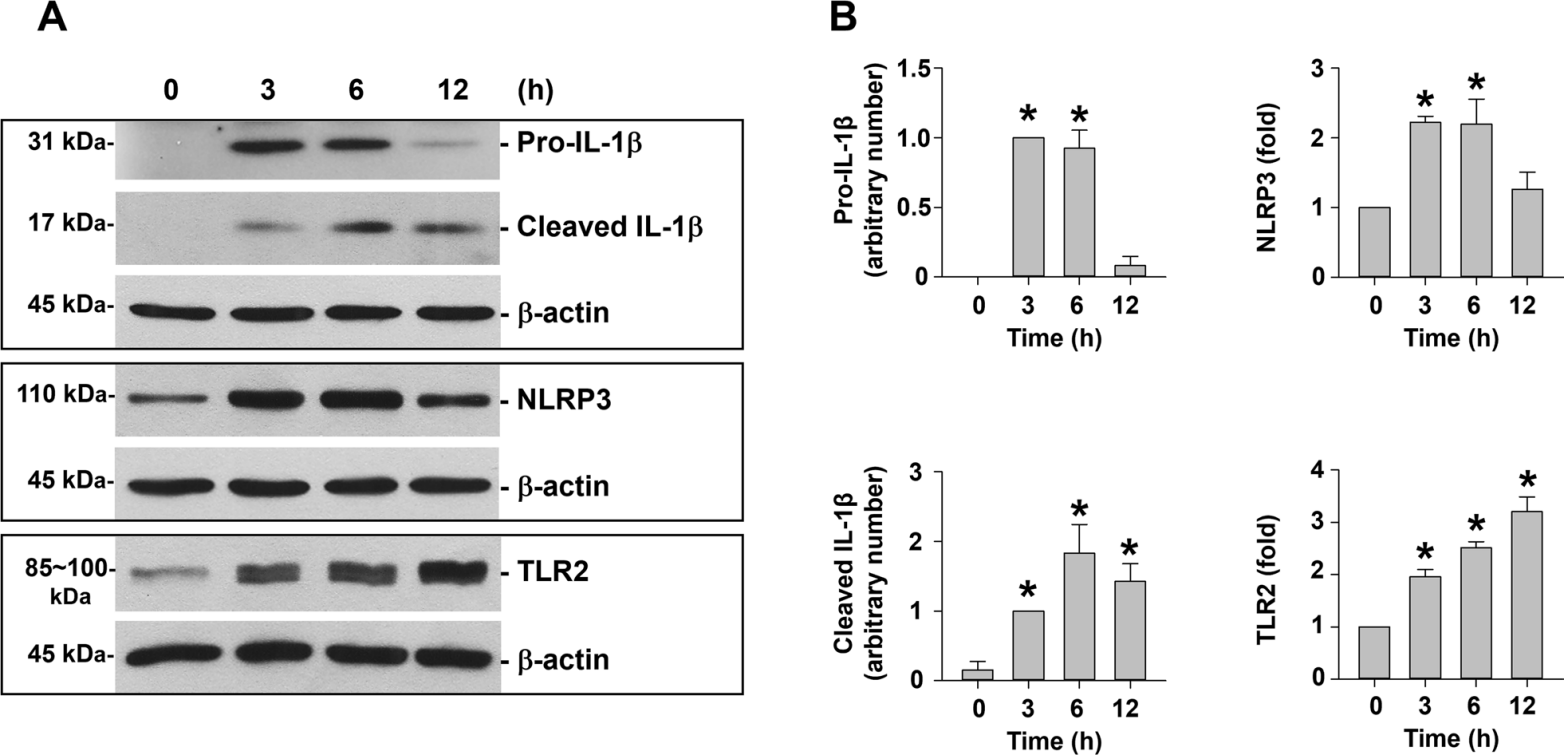
Time-response study of protein expression of IL-1β, NLRP3, and TLR2 in MH-S cells exposed to PM_2.5_. The cells were treated with 50 μg/mL of PM_2.5_ for 3, 6, and 12 h. The cells treated with physiological saline were used as control. **a** is the Western blot result of a single experiment while **b** is the normalized results of Western blots. Data are shown as mean ± SEM (*n* = 3). * *p* < 0.05 vs. control

The upregulation of IL-1β, NLRP3, and TLR2 after PM_2.5_ exposure was further confirmed at the protein level by Western blot. The results of the dose–response study revealed that exposure of MH-S cells to 25, 50, and 100 μg/mL of PM_2.5_ for 6 h caused significant upregulation of pro-IL-1β, cleaved IL-1β, NLRP3, and TLR2 (Fig. 3a, b). The time-response study showed that both pro-IL-1β and cleaved IL-1β were significantly increased in MH-S cells exposed to 50 μg/mL of PM_2.5_ for 3 h and 6 h after exposure (Fig. 4a, b). Then the pro-IL-1β level decreased to near baseline level after 50 μg/mL of PM_2.5_ treatment for 12 h while the cleaved IL-1β level was still significantly increased (Fig. 4a, b). NLRP3 protein expression level had a similar trend as pro-IL-1β; increased at 3 and 6 h after 50 μg/mL of PM_2.5_ treatment and decreased at 12 h after treatment (Fig. 4a, b). The TLR2 protein level was significantly increased in MH-S cells exposed to 50 μg/mL of PM_2.5_ for 3, 6, and 12 h (Fig. 4a, b).

### High glucose pretreatment enhanced PM_2.5_-induced upregulation of IL-1β and NLRP3, but not TLR2

**Fig. 5:**
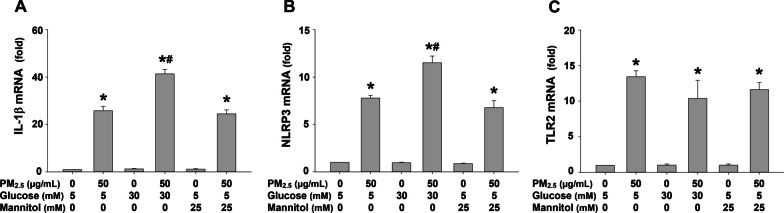
Enhanced mRNA expression of IL-1β (**a**) and NLRP3 (**b**), but not TLR2 (**c**) in MH-S cells exposed to PM_2.5_ at high glucose setting. The cells were treated with 50 μg/mL of PM_2.5_ for 3 h with/without 30 mM of glucose pretreatment for 18 days. Mannitol was used as an osmolality control. The cells treated with physiological saline were used as control. The mRNA expression of the gene was determined by RT-qPCR and normalized to the β-actin expression in the same sample. Data are shown as mean ± SEM (*n* = 3). * *p* < 0.05 vs. control; ^#^ *p* < 0.05 vs. group with PM_2.5_ treatment only

At first, the mRNA expression levels of IL-1β, NLRP3, and TLR2 were determined by RT-qPCR after MH-S cells were pretreated with 30 mM of glucose for 9 days followed by treatment with 50 μg/mL of PM_2.5_ for 3 h. The results showed that although PM_2.5_ exposure significantly upregulated IL-1β, NLRP3, and TLR2 in control cells (5 mM glucose), no enhanced expression was observed in the cells with high glucose pretreatment (data not shown). Then the cells were maintained in 30 mM of glucose environment for 18 days followed by treatment with 50 μg/mL of PM_2.5_ for 3 h. Our results showed that PM_2.5_ exposure caused significantly increased mRNA expression levels of IL-1β, NLRP3, and TLR2 (Fig. 5), which were consistent with the results of dose- and time-dependent studies (Fig. 2). Moreover, the mRNA levels of both IL-1β and NLRP3, but not TLR2, were enhanced in MH-S cells with high glucose, but not osmolarity control mannitol, pretreatment (Fig. 5a-c). Thus, a time point of 18 days of high glucose pretreatment was selected. The enhanced mRNA expression of IL-1β and NLRP3 by PM_2.5_ exposure with high glucose pretreatment was also observed in primary alveolar macrophages obtained from C57BL/6J mice by bronchoalveolar lavage (BAL) (Additional file 2).

**Fig. 6:**
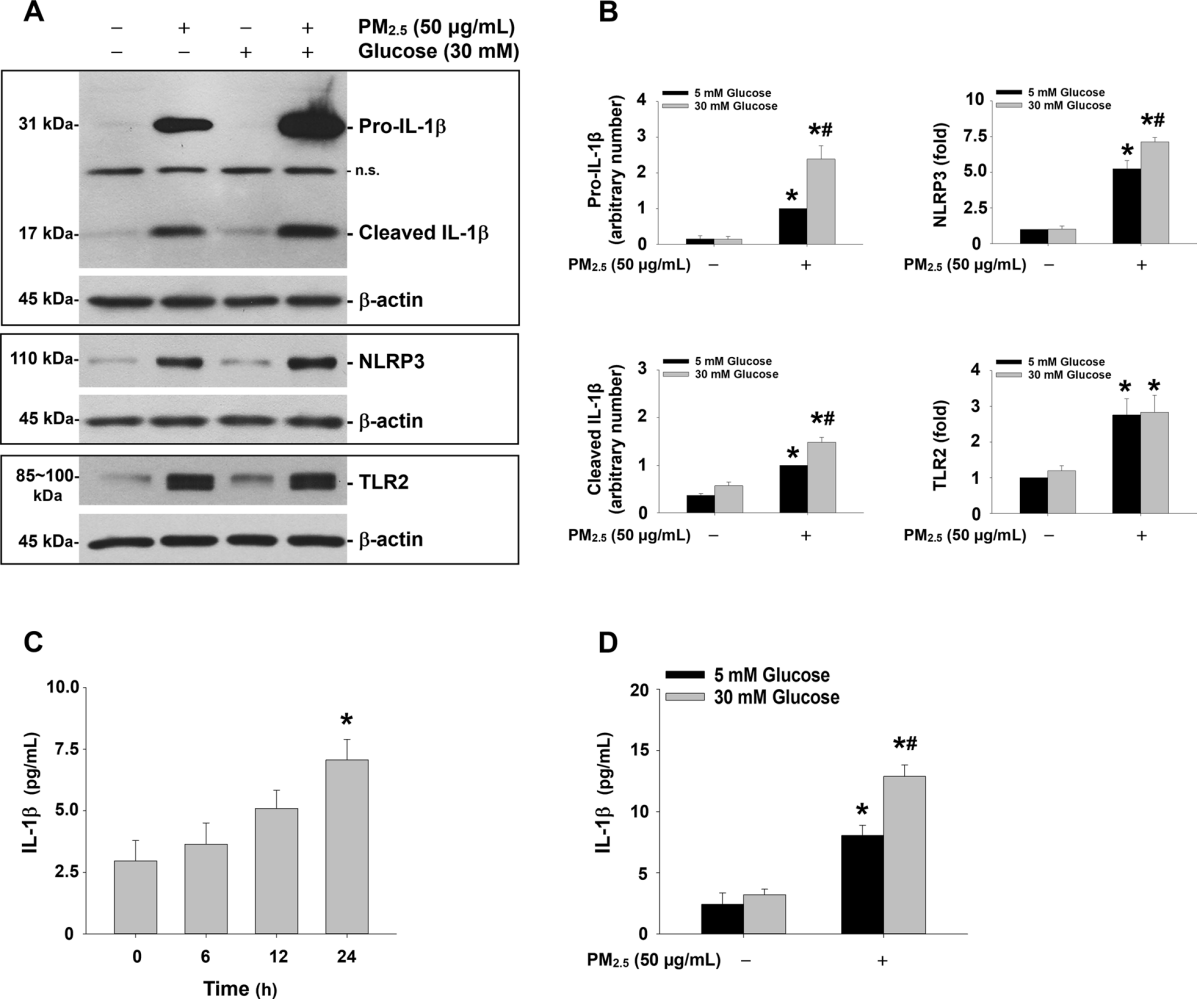
High glucose pretreatment enhanced the effects of PM_2.5_ on the activation of NLRP3 inflammasome, but not TLR2, in MH-S cells. The cells were treated with 50 μg/mL of PM_2.5_ for 6 h (**a** and **b**) or 24 h (**d**) with/without 30 mM of glucose pretreatment for 18 days. **c** The cells were treated with 50 μg/mL of PM_2.5_ for 6, 12, and 24 h. The cells treated with physiological saline were used as control. **a** is the Western blot result of a single experiment while **b** is the normalized results of Western blots. **c** and **d** The cell culture media were collected for ELISA by Mouse IL-1beta ELISA kit (Invitrogen). Data are shown as mean ± SEM (*n* = 3). * *p* < 0.05 vs. control; ^#^ *p* < 0.05 vs. group with PM_2.5_ treatment only. n.s., not specific

The mRNA expression results were further confirmed at protein levels by Western blot; the protein expression levels of pro-IL-1β, cleaved IL-1β, and NLRP3, but not TLR2, were enhanced in MH-S cells exposed to 50 μg/mL of PM_2.5_ for 6 h with high glucose pretreatment for 18 days (Fig. 6a, b). Moreover, the IL-1β protein levels in the cell culture media, determined by ELISA, were increased in a time-dependent manner when MH-S cells were exposed to 50 μg/mL of PM_2.5_ for 6, 12, and 24 h, with significant increase at 24 h after PM_2.5_ exposure (Fig. 6c). And this effect was enhanced in the cells with 30 mM glucose pretreatment for 18 days (Fig. 6d). These results suggest that in a high glucose environment, PM_2.5_ exposure caused enhanced production of pro-inflammatory cytokine IL-1β and activation of NLRP3 inflammasome.

### Enhanced NF-κB nuclear translocation in MH-S cells exposed to PM_2.5_ in a high glucose environment

**Fig. 7:**
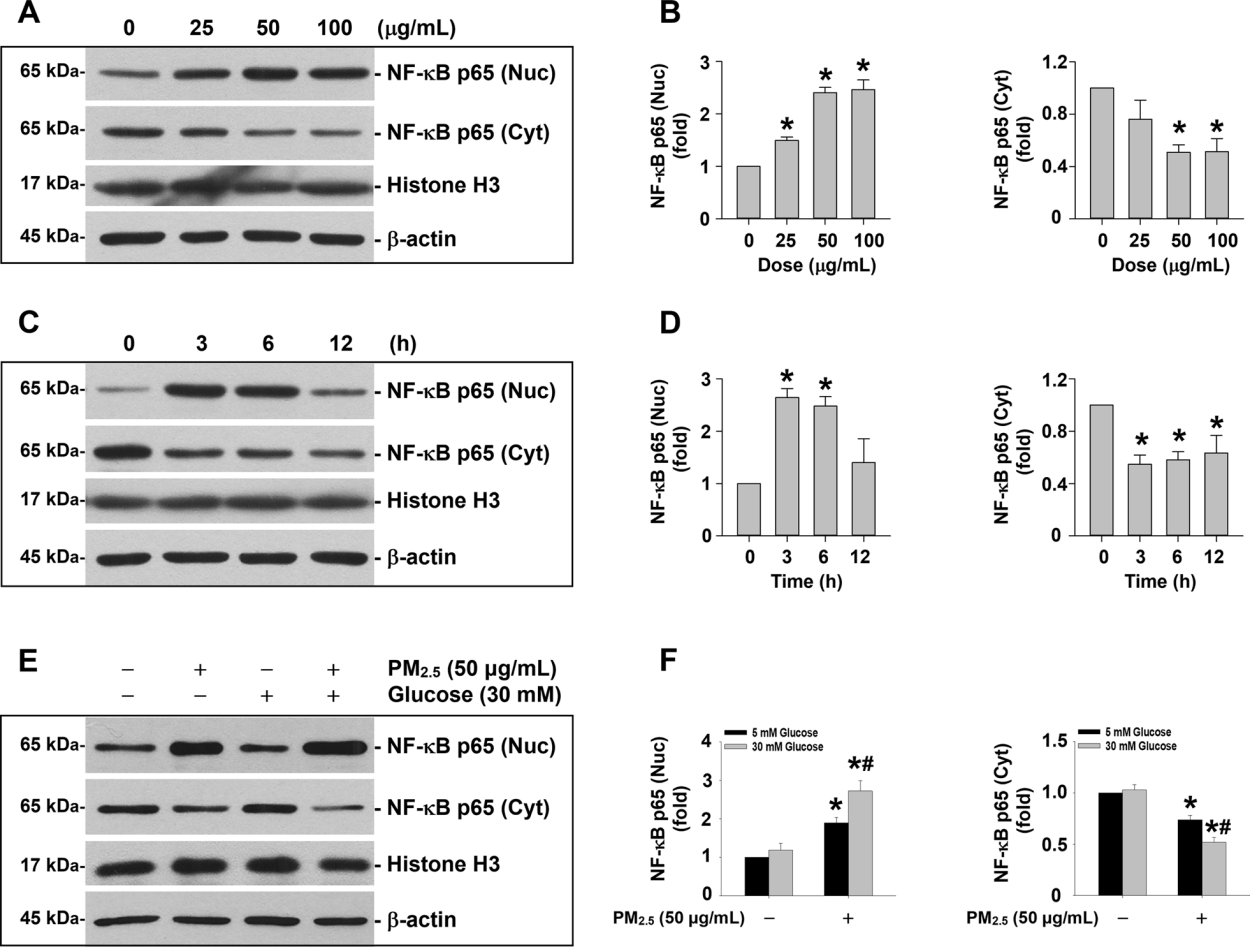
Enhanced NF-κB nuclear translocation in MH-S cells exposed to PM_2.5_ in a high glucose environment. The cells were treated with 25, 50, and 100 μg/mL of PM_2.5_ for 6 h (**a** and **b**) or with 50 μg/mL of PM_2.5_ for 3, 6, and 12 h (**c** and** d)**. **e** and **f** The cells were treated with 50 μg/mL of PM_2.5_ for 6 h with/without 30 mM glucose pretreatment for 18 days. The cells treated with physiological saline were used as control. Nuclear and cytoplasmic proteins were extracted from the cells by using NE-PER™ Nuclear and Cytoplasmic Extraction Reagent (Thermo Scientific). The expression of β-actin was used as an internal reference for cytoplasmic protein while histone H3 was for nuclear protein. **a, c, e** are Western blot results of a single experiment while **b, d, f** are normalized results of Western blots. Data are shown as mean ± SEM (*n* = 3). * *p* < 0.05 vs. control; ^#^ *p* < 0.05 vs. group with PM_2.5_ treatment only. Nuc, nuclear protein; Cyt, cytoplasmic protein

NF-κB binding sites have been identified in the promoter region of both human and mouse IL-1β [25–27] and NLRP3 [28, 29]. Therefore, we next determined whether PM_2.5_ exposure caused NF-κB nuclear translocation by detection of both nuclear and cytoplasmic NF-κB p65 protein levels by Western blot. The results of both dose- and time-response studies showed that exposure of MH-S cells to PM_2.5_ caused significantly increased expression of NF-κB p65 in the nuclei but decreased expression in the cytoplasm, indicating that PM_2.5_ exposure caused NF-κB nuclear translocation (Fig. 7a-d). After MH-S cells were pretreated with 30 mM of glucose for 18 days, 50 μg/mL of PM_2.5_ treatment caused an even higher level of nuclear and lower level of cytoplasmic NF-κB p65 protein expression (Fig. 7e, f), indicating PM_2.5_ exposure caused enhanced nuclear translocation of NF-κB in a high glucose environment.

### Enhanced ROS generation in MH-S cells exposed to PM_2.5_ at high glucose setting and the possible source of ROS

**Fig. 8:**
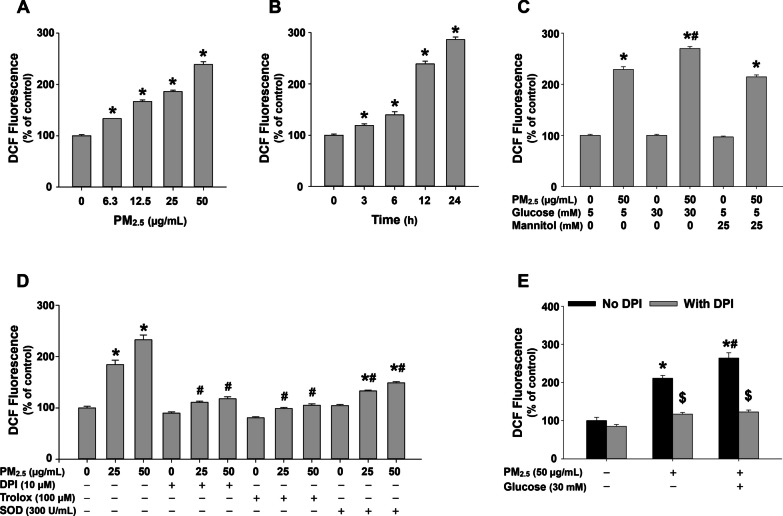
Enhanced ROS generation in MH-S cells exposed to PM_2.5_ with high glucose pretreatment. The cells were pretreated with 5 µM of H_2_DCF-DA for 2 h, followed by treatment with PM_2.5_ for 12 h (**a**) or with 50 μg/mL of PM_2.5_ for 3, 6, 12, and 24 h (**b**). **c** The cells were pretreated with 30 mM of glucose for 18 days and 5 µM of H_2_DCF-DA for 2 h, followed by treatment with PM_2.5_ for 12 h. Mannitol was used as an osmolality control. **d** The cells were pretreated with ROS inhibitors or scavengers for 2 h, followed by treatment with 5 µM of H_2_DCF-DA for 2 h and PM_2.5_ for 12 h. **e** The cells were pretreated with 30 mM of glucose for 18 days and DPI for 2 h, followed by treatment with 5 µM of H_2_DCF-DA for 2 h and PM_2.5_ for 12 h. The cells treated with physiological saline were used as control. Data are shown as mean ± SEM (*n* = 3). * *p* < 0.05 vs. control; ^#^ *p* < 0.05 vs. group with the same dose of PM_2.5_ treatment only; ^$^ *p* < 0.05 vs. group with the same PM_2.5_ and glucose treatments, but without DPI pretreatment

ROS generation in mouse alveolar macrophages MH-S after exposure to PM_2.5_ with/without high glucose pretreatment was determined by measurement of DCF fluorescence intensity. PM_2.5_ exposure caused a dose-and a time-dependent increase in DCF fluorescence when the cells were exposed to 6.3, 12.5, 25, and 50 μg/mL of PM_2.5_ for 12 h (Fig. 8a) or to 50 μg/mL of PM_2.5_ for 3, 6, 12, and 24 h (Fig. 8b). When the cells were pretreated with 30 mM of glucose for 18 days, 50 μg/mL of PM_2.5_ exposure induced an enhanced ROS generation (Fig. 8c). However, 30 mM glucose treatment alone did not cause any increase in ROS generation, and the osmolality control mannitol did not cause any enhanced ROS generation induced by PM_2.5_ exposure (Fig. 8c). These results indicated that PM_2.5_ exposure could induce enhanced oxidative stress on alveolar macrophages in a high glucose environment.

To determine the possible source of ROS in MH-S cells exposed to PM_2.5_ with/without high glucose pretreatment, ROS inhibitors or scavengers were used to pretreat the cells prior to 25 and 50 μg/mL of PM_2.5_ exposure. The results showed that pretreatment of the cells with 10 μM of DPI (a specific NADPH oxidase inhibitor), 100 μM of Trolox (an antioxidant), or 300 U/mL of superoxide dismutase (SOD, an enzyme that catalyzes the dismutation of the superoxide radical into oxygen and hydrogen peroxide) significantly inhibited PM_2.5_-induced ROS generation (Fig. 8d). However, the production of superoxide by mitochondria was not increased after PM_2.5_ exposure by using MitoSOX™ Red Mitochondrial Superoxide Indicator (Additional file 3). In addition, pretreatment of the cells with 10 μM of DPI also abolished PM_2.5_-induced ROS generation in the cells in a high glucose environment (Fig. 8e). All these results suggest that NADPH oxidase, rather than mitochondria, may be responsible for PM_2.5_-induced enhanced ROS generation in alveolar macrophages with high glucose pretreatment.

**Fig. 9:**
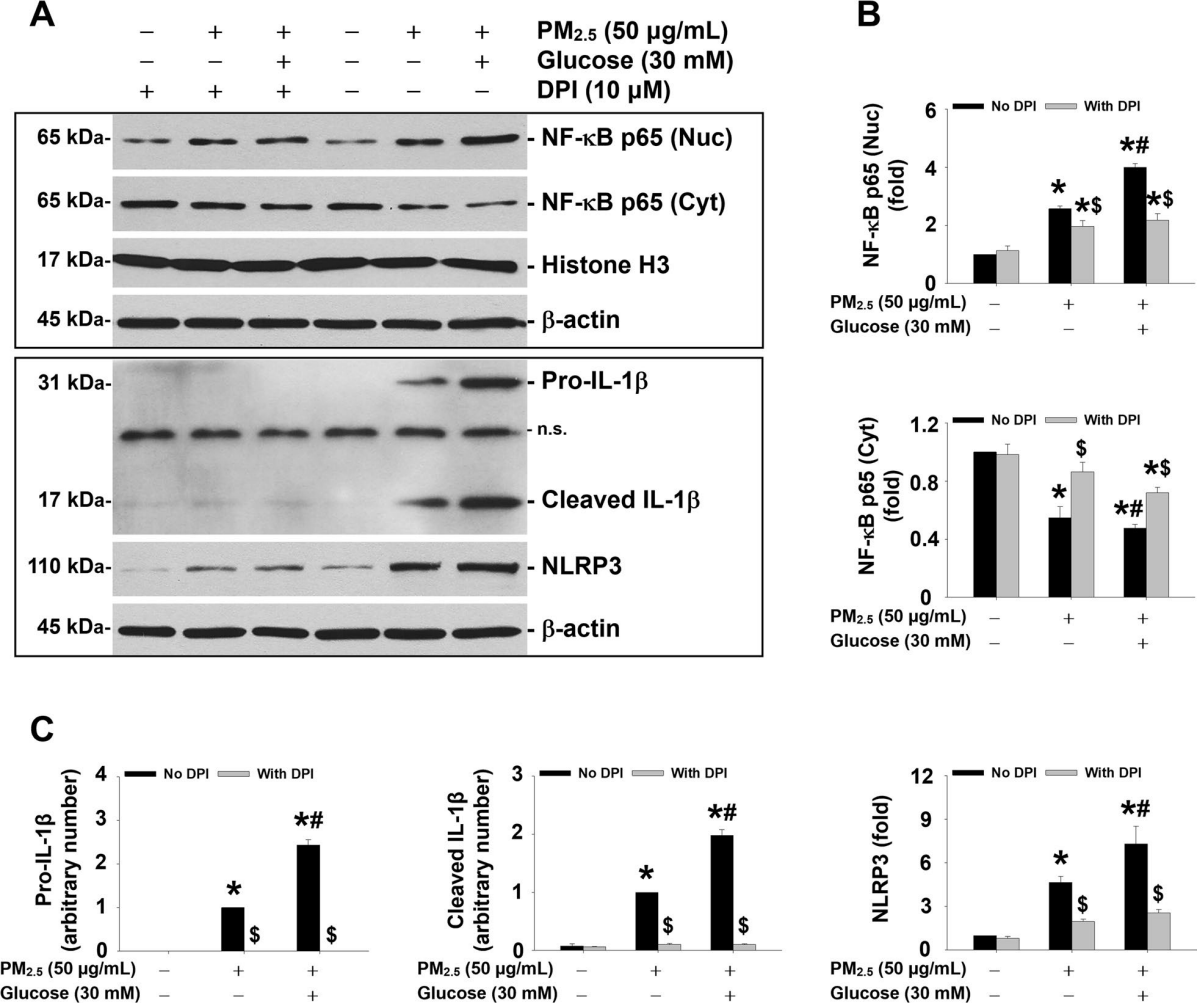
Inhibition of NADPH oxidase by DPI abolished PM_2.5_-induced NF-κB nuclear translocation, NLRP3 inflammasome activation, and IL-1β production. The MH-S cells were treated with 10 μM DPI for 2 h, followed by 50 μg/mL of PM_2.5_ for 6 h with/without 30 mM glucose pretreatment for 18 days. The cells treated with physiological saline were used as control. Nuclear and cytoplasmic proteins were extracted from the cells by using NE-PER™ Nuclear and Cytoplasmic Extraction Reagent (Thermo Scientific). The expression of β-actin was used as an internal reference for cytoplasmic protein while histone H3 was for nuclear protein. **a** is the Western blot result of a single experiment while **b** and** c** are the normalized results of Western blots. Data are shown as mean ± SEM (*n* = 3). * *p* < 0.05 vs. control; ^#^ *p* < 0.05 vs. group with PM_2.5_ treatment only; ^$^ *p* < 0.05 vs. group with the same PM_2.5_ and glucose treatments, but without DPI pretreatment. Nuc, nuclear protein; Cyt, cytoplasmic protein; n.s., not specific

In addition, to determine the role of activation of NADPH oxidase in NF-κB nuclear translocation, NLRP3 inflammasome activation, and IL-1β production induced by PM_2.5_ exposure in a high glucose environment, the cells with/without high glucose pretreatment were treated with 10 μM of DPI for 2 h followed by treatment with 50 μg/mL of PM_2.5_ for 6 h. The results showed that DPI pretreatment significantly attenuated PM_2.5_-induced NF-κB nuclear translocation and increased expression of NLRP3, pro-IL-1β, and cleaved IL-1β in MH-S cells with/without high glucose pretreatment (Fig. 9a-c), suggesting ROS generated by NADPH oxidase is responsible for PM_2.5_-induced NF-κB nuclear translocation, NLRP3 inflammasome activation, and IL-1β production in alveolar macrophages.

### Enhanced MMP-9 production by IL-1β in alveolar macrophages exposed to PM_2.5_ in a high glucose environment

**Fig. 10:**
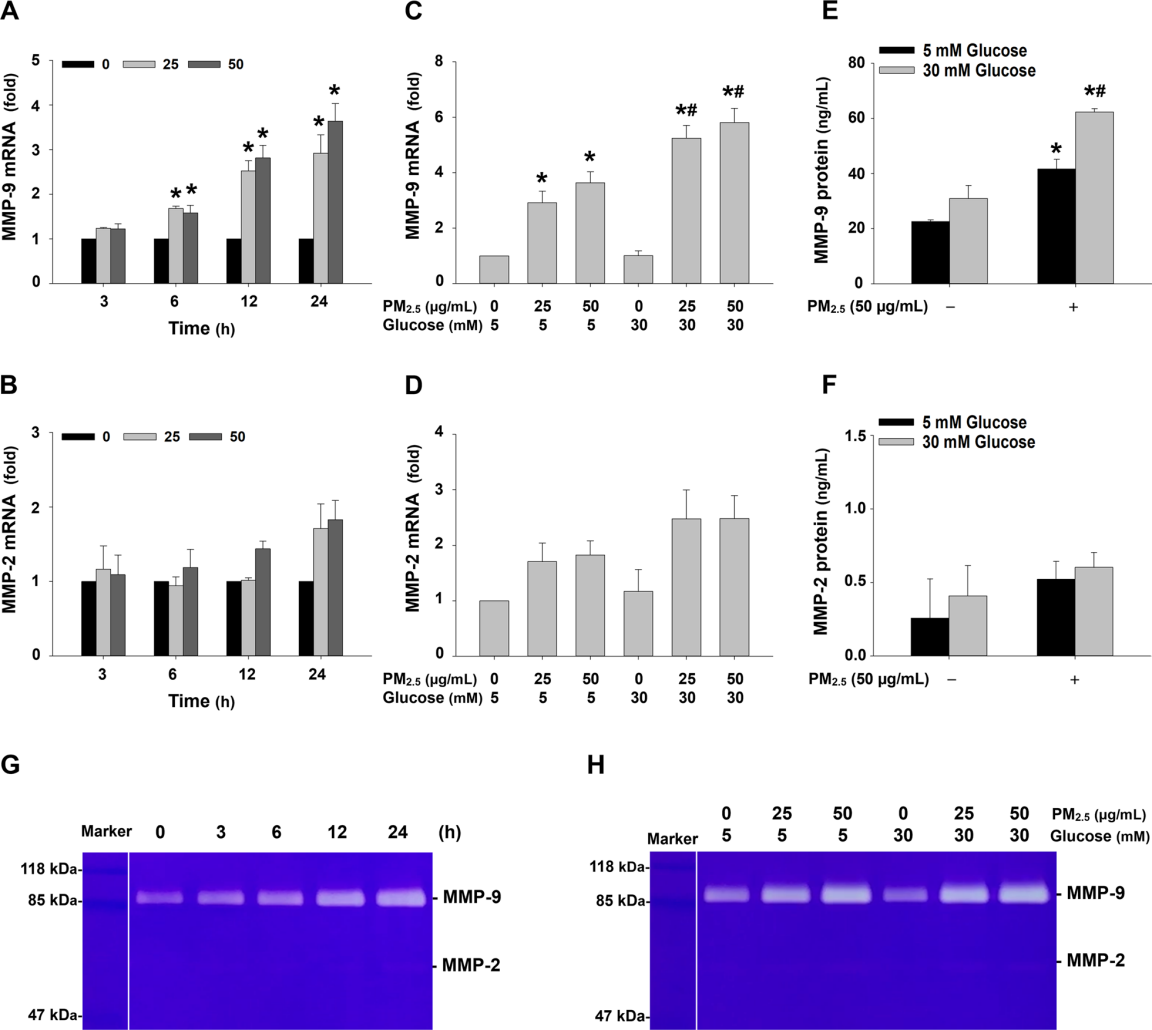
The expression levels and enzymatic activities of MMP-2 and MMP-9 in MH-S cells exposed to PM_2.5_ with/without high glucose pretreatment. The cells were treated with 25 or 50 μg/mL of PM_2.5_ for 3, 6, 12, and 24 h (**a, b, g**) or 24 h (**c-f, h**) with/without 30 mM of glucose pretreatment for 18 days. The cells treated with physiological saline were used as control. **a**-**d** The mRNA expressions of MMP-2 and MMP-9 were determined by RT-qPCR and normalized to the β-actin expression in the same sample. **e, f** The protein levels of MMP-2 and MMP-9 in cell culture media were determined by ELISA. Data are shown as mean ± SEM (*n* = 3). * *p* < 0.05 vs. control; ^#^ *p* < 0.05 vs. group with the same dose of PM_2.5_ treatment only. **g, h** The enzymatic activities of MMP-2 and MMP-9 in the cell culture media were determined by gelatin zymography assay

At first, MMP-2 and MMP-9 mRNA expression levels were determined by RT-qPCR in MH-S cells exposed to 25 or 50 μg/mL of PM_2.5_ for 3, 6, 12, and 24 h. The results showed that exposure of MH-S cells to PM_2.5_ caused a time-dependent and significant increase in MMP-9 mRNA expression (Fig. 10a). However, PM_2.5_ exposure only caused a slight, but not significant, upregulation of MMP-2 (Fig. 10b), TIMP-1, and TIMP-2 (Additional file 4). Since significantly increased IL-1β protein was detected in the cell culture medium after 24 h of PM_2.5_ treatment (Fig. 6c), and previous studies demonstrated that IL-1β can induce MMP-9 upregulation [30–33], a time point of 24 h was selected for MMPs assessment. When the cells were pretreated with 30 mM of glucose for 18 days followed by PM_2.5_ exposure for 24 h, the mRNA expression level of MMP-9, but not MMP-2, was significantly enhanced (Fig. 10c, d). MMP-2 and MMP-9 protein levels were determined in the cell culture media by ELISA. The results showed that 50 μg/mL of PM_2.5_ exposure for 24 h caused a significant increase in MMP-9, but not MMP-2, protein level in the cell culture media, and pretreatment with high glucose enhanced this effect (Fig. 10e, f). The results of MMP-9 enzymatic activity determined by gelatin zymography assay had a similar trend as MMP-9 mRNA and protein expression (Fig. 10g, h).

**Fig. 11:**
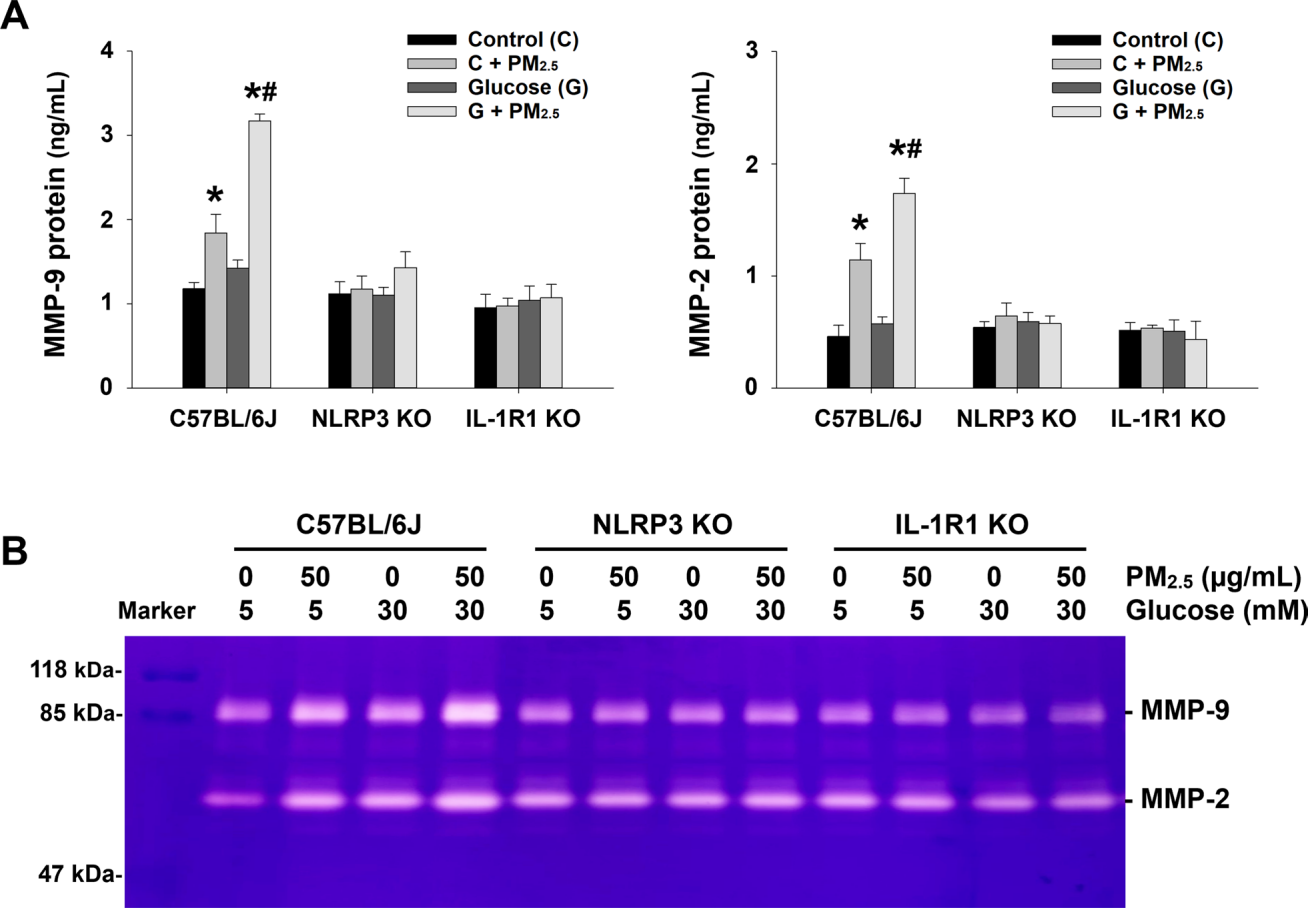
The protein levels and enzymatic activities of MMP-2 and MMP-9 in the cell culture media of primary mouse alveolar macrophages exposed to PM_2.5_ with/without high glucose pretreatment. Primary mouse alveolar macrophages were obtained from C57BL/6J, NLRP3 KO, and IL-1R1 KO mice, respectively, by bronchoalveolar lavage (BAL) and pretreated with 30 mM of glucose for 24 h prior to treatment with 50 μg/mL of PM_2.5_ for another 24 h. The cells treated with physiological saline were used as control. **a** The protein levels of MMP-2 and MMP-9 in the cell culture media were determined by ELISA. Data are shown as mean ± SEM of three independent experiments. * *p* < 0.05 vs. control; ^#^ *p* < 0.05 vs. the group with PM_2.5_ treatment only. **b** The enzymatic activities of MMP-2 and MMP-9 in the cell culture media were determined by gelatin zymography assay

To determine whether enhanced MMP-9 expression and enzymatic activity in alveolar macrophages exposed to PM_2.5_ with high glucose pretreatment was induced by enhanced IL-1β level with PM_2.5_ and high glucose exposure, primary alveolar macrophages isolated from NLRP3 KO and IL-1R1 KO mice were used. Knocking out of NLRP3 results in malfunction of NLRP3 inflammasome, thus preventing the cleavage of pro-IL-1β from producing mature IL-1β [34] while IL-1R1 KO mice fail to respond to IL-1 [35]. Primary alveolar macrophages isolated from wild-type C57BL/6J mice were used as control. The results showed that both MMP-2 and MMP-9 protein levels and enzymatic activities in the cell culture media of primary alveolar macrophages obtained from wild-type C57BL/6J mice were significantly increased when the cells were treated with 50 μg/mL of PM_2.5_ for 24 h, and this effect was enhanced when the cells were pretreated with 30 mM of glucose for 24 h, which were consistent with the results from MH-S cells (Fig. 11). However, PM_2.5_ exposure with/without high glucose pretreatment did not cause significant increases in MMP-2 and MMP-9 protein levels and enzymatic activitiesin primary alveolar macrophages obtained from NLRP3 KO and IL-1R1 KO mice (Fig. 11). Our results demonstrated that enhanced MMP production was through IL-1β in alveolar macrophages exposed to PM_2.5_ with high glucose pretreatment.

## Discussion

**Fig. 12:**
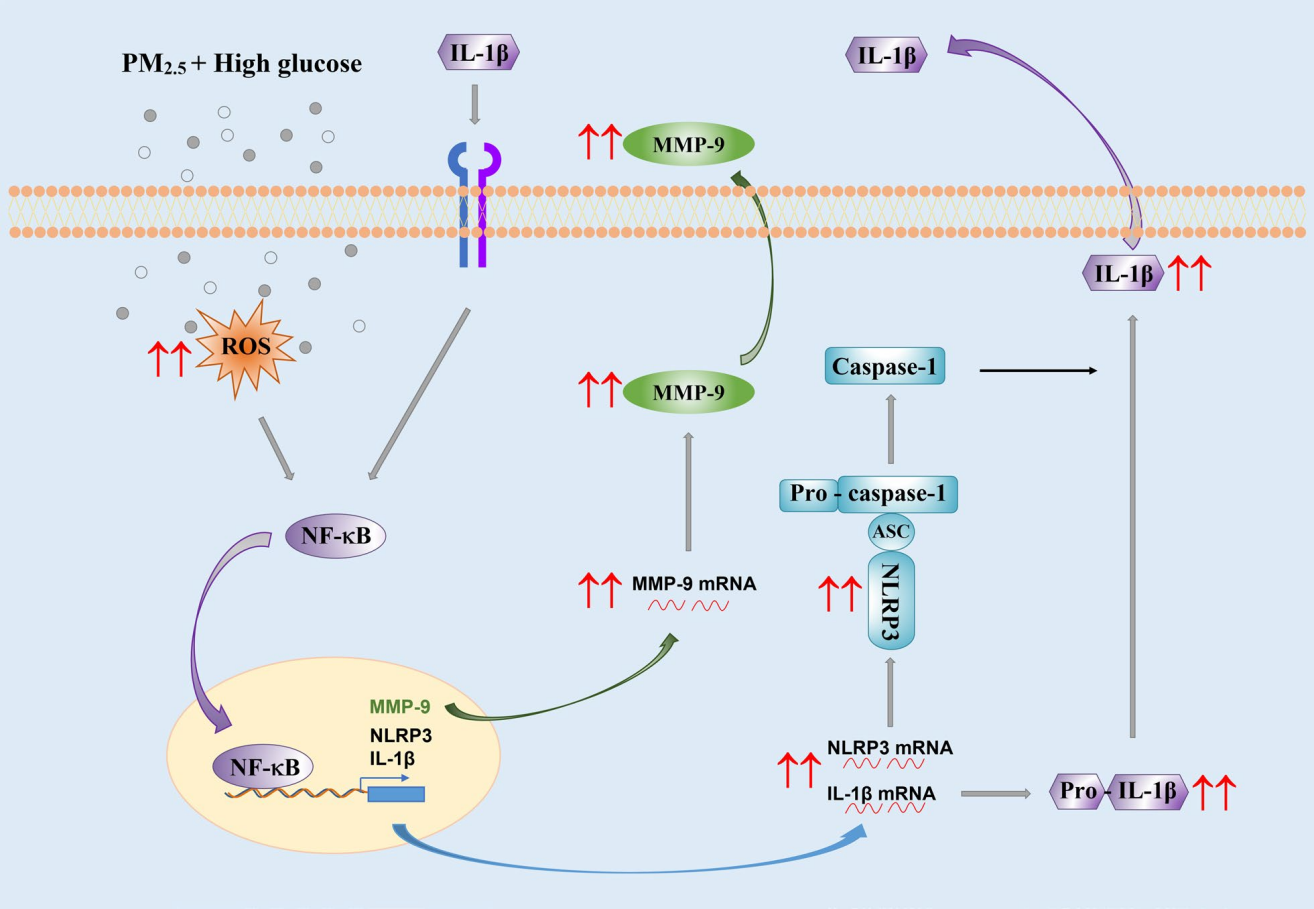
Schematic diagram of the possible mechanisms involved in the enhanced production of pro-inflammatory cytokine IL-1β and increased MMP-9 activity in alveolar macrophages exposed to PM_2.5_ in a high glucose environment. PM_2.5_ exposure in a high glucose environment causes enhanced ROS generation, which induces nuclear translocation of the transcription factor NF-κB. Binding of NF-κB on the promoter regions of NLRP3 and IL-1β results in their upregulation, leading to the activation of NLRP3 inflammasome, which cleaves pro-IL-1β to IL-1β. IL-1β is involved in the inflammatory response and a variety of cellular activities including MMP-9 upregulation

In this study, the effects of PM_2.5_ on mouse alveolar macrophages in a high glucose environment were explored. Specifically, we investigated whether activation of NLRP3 inflammasome was involved in the increased susceptibility of alveolar macrophages to PM_2.5_ with high glucose pretreatment and the potential underlying mechanisms (Fig. 12). Our results demonstrated that exposure of alveolar macrophages to non-cytotoxic doses of PM_2.5_ led to upregulation of pro-inflammatory cytokine IL-1β, activation of NLRP3 inflammasome, increased nuclear translocation of NF-κB, increased generation of ROS, and increased expression and enzymatic activity of MMP-9, all of which were enhanced when cells were pretreated with high glucose. In addition, these enhanced effects were abolished when cells were pretreated with DPI, an NADPH oxidase specific inhibitor, and increased MMP-9 activity was not observed in primary alveolar macrophages obtained from NLRP3 KO and IL-1R1 KO mice. Moreover, although exposure of alveolar macrophages to PM_2.5_ also caused TLR2 upregulation, treatment with PM_2.5_ with high glucose pretreatment had no enhanced effects on TLR2 expression. These results suggest that enhanced production of pro-inflammatory cytokine IL-1β in alveolar macrophages exposed to PM_2.5_ with high glucose pretreatment may be through activation of NLRP3 inflammasome and nuclear translocation of NF-κB, which are due to oxidative stress induced by PM_2.5_ and high glucose, with NADPH oxidase being the main source of ROS generated by PM_2.5_ exposure in a high glucose environment. In addition, enhanced production of IL-1β may be necessary for the enhanced expression and enzymatic activity of MMP-9.

At first, the cytotoxicity of PM_2.5_ and/or glucose in alveolar macrophages MH-S was determined by both MTS assay and alamarBlue™ assay. A dose-dependent cytotoxic effect was observed when cells were exposed to PM_2.5_ at concentrations ranging from 0 to 400 µg/mL. Exposure to 100 µg/mL and above of PM_2.5_ caused significant cytotoxicity in MH-S cells while concentrations of 50 µg/mL and less did not. Thus, to avoid the confounding effects caused by cytotoxicity, non-cytotoxic doses of PM_2.5_ (≤ 50 µg/mL) were selected for the following mechanism studies. Currently, the primary “annual average concentration” standard set by the US EPA for PM_2.5_ is 12 µg/m^3^ [36]. However, PM_2.5_ concentrations exceed this standard in many regions in the United States and around the world. In Three Rivers, California, the most PM_2.5_ polluted regional city in the United States as of 2021, the annual average is 27.4 μg/m^3^ [37]. High concentrations of PM_2.5_ worldwide can be found in Asia, Africa, and the Middle East, reaching nearly 100 μg/m^3^ in some regional cities [37]. Exposure of MH-S cells to glucose up to 50 mM did not cause any cytotoxicity. Thus, in this study, 30 mM glucose was selected to mimic high blood glucose level in diabetic individuals while 5 mM glucose was chosen as the control. Normal fasting glucose level was defined as < 200 mg/dL (11.1 mM) in mice and < 100 mg/dL (5.6 mM) in humans [38].

Alveolar macrophages are the first line of defense against inhaled particles. Previous studies have shown that diabetic patients have elevated glucose concentration in airway surface liquid (ASL). For example, Baker et al. reported that the glucose concentration in ASL, as reflected by breath glucose, was increased in diabetic patients as compared with that in the healthy volunteers, which was even more significant in the diabetic patient with airway inflammation, such as cystic fibrosis-related diabetes (CFRD) [39]. In addition, increased glucose level in the BALF was also observed in animal models. For example, the glucose concentration was found to be significantly higher in the BALF from hyperglycemic db/db or streptozotocin-induced hyperglycemic mice as compared with that in the nondiabetic control mice [40–42]. Higher glucose level in BALF was also observed in alloxan-induced hyperglycemic rats [43]. Thus, alveolar macrophages are likely in a high glucose environment in individuals with diabetes. In addition, alveolar macrophages are derived from adult blood monocytes or embryonic/fetal precursors [44]. During lung injury induced by environmental factors such as PM_2.5_ exposure, increased blood monocyte-derived macrophages pool in the lungs. For example, a previous study showed that herpes virus infection in mouse lungs promoted an alveolar macrophage compartment mainly consisting of blood monocyte-derived macrophages [45]. In diabetic individuals, these blood monocyte-derived macrophages will have been exposed to a high-glucose environment before they migrated into the lungs. In this study, 30 mM glucose was used to pretreat cells. Although the blood glucose in diabetic individuals will not remain at such high levels, it is not uncommon for the blood glucose level to reach as high as 30 mM in uncontrolled diabetes and patients with diabetic ketoacidosis or hyperosmolar hyperglycemic state (HHS). Such high glucose concentration was also widely used in previous studies.

Previous studies have shown that PM induces the production of pro-inflammatory cytokines and other inflammatory mediators in many cell types [46, 47]. Exposure to high glucose also resulted in the production of cytokines such as IL-1β in human monocytes [48]. IL-1β is a pro-inflammatory cytokine thought to be involved in the initiation of the inflammatory process and thus contributes to acute and chronic inflammation [13, 14]. IL-1β is not expressed in a steady state but is strongly inducible following pro-inflammatory signals [49]. IL-1β is also involved in the pathogenesis of diabetes mellitus. Elevated levels of IL-1β, IL-6, and C-reactive protein (CRP) are predictive of T2D [50–53]. In newly diagnosed T1D patients, IL-1β is increased and likely acts as an early inflammatory signal in T1D progression [54]. In the present study, increased expression of both pro-IL-1β and cleaved IL-1β was observed in alveolar macrophages exposed to PM_2.5_, which was enhanced when cells were pretreated with high glucose prior to PM_2.5_ exposure, suggesting that individuals with diabetes may be more vulnerable to PM-induced pulmonary and systemic inflammation and other PM-related health problems.

Many factors may cause IL-1β upregulation, including activation of NLRP3 inflammasome and/or toll-like receptors (TLRs), oxidative stress, etc. [15–17]. NLRP3 inflammasome is a cytoplasmic multiprotein complex that is currently the most extensively studied inflammasome, and is assembled by the recruitment of adapter protein ASC and pro-caspase-1 by NLRP3 protein when cells sense intracellular danger signals [14, 15]. NLRP3 inflammasome mediates the cleavage and activation of caspase-1, which cleaves pro-IL-1β to active IL-1β [14, 15]. In this study, both mRNA and protein expression levels of NLRP3 in MH-S cells exposed to PM_2.5_ with/without high glucose pre-treatment were assessed. The results showed that both mRNA and protein expressions of NLRP3 were upregulated by PM_2.5_ exposure, with enhanced expression when cells were pre-treated with high glucose, suggesting that the activation of NLRP3 inflammasome may be involved in enhanced production of IL-1β in alveolar macrophages in a high glucose environment after PM_2.5_ exposure.

In addition to NLRP3 upregulation, increased mRNA expression of IL-1β and increased protein expression of pro-IL-1β were also observed in alveolar macrophages with PM_2.5_ exposure with/without high glucose pretreatment. NF-κB could be responsible for the upregulation of IL-1β and NLRP3 [25, 26, 28, 29]. NF-κB binding sites have been identified in the promoter regions of both human and mouse IL-1β [25–27] and NLRP3 [28, 29]. NF-κB signaling is involved in a large array of immune and inflammatory responses and diseases [55]. The best-studied and most important NF-κB family member is RelA (also named p65). In this study, NF-κB p65 was found to be translocated from the cytoplasm to the nucleus upon PM_2.5_ exposure, and this effect was enhanced when the cells were pretreated with high glucose, indicating NF-κB was involved in the upregulation of IL-1β and NLRP3 after PM_2.5_ exposure in the high glucose environment. Many factors including TLRs [29], oxidative stress [56, 57], pro-inflammatory cytokines such as IL-1β [58], etc. can activate NF-κB. TLR2, but not TLR4, was observed to be activated by PM_2.5_ exposure, but no enhanced activation of TLR2 was observed after cells were pretreated with high glucose. However, ROS was enhanced when cells were treated with PM_2.5_ in a high glucose environment, indicating NF-κB activation mediated by ROS, not TLRs, is responsible for enhanced PM_2.5_-induced upregulation of NLRP3 and IL-1β in a high glucose environment.

Oxidative stress is considered an important mechanism underlying PM-induced health effects. Increased ROS generation in cells exposed to PM and activation of ROS-responsive transcription factors such as NF-κB and AP-1, which, together with the depletion of antioxidant defenses, can lead to the release of pro-inflammatory cytokines such as IL-1β [56, 57]. Here, ROS generation in MH-S cells exposed to PM_2.5_ with/without high glucose pre-treatment was determined. The results showed that ROS generation was increased by PM_2.5_ exposure, which was enhanced when cells were in a high glucose environment. Pretreatment of alveolar macrophages with an NADPH oxidase inhibitor, DPI, inhibited PM_2.5_-induced ROS generation, but mitochondria superoxide specific detector, MitoSOX, failed to detect significant superoxide generation after PM_2.5_ exposure, suggesting that the ROS induced by PM_2.5_ exposure were mainly generated by NADPH oxidase rather than through the mitochondria. Moreover, pretreatment of alveolar macrophages with DPI attenuated NF-κB nuclear translocation and upregulation of NLRP3 and IL-1β induced by PM_2.5_ exposure in a high glucose environment, confirming that enhanced IL-1β production upon PM_2.5_ exposure is through NLRP3 inflammasome activation and NF-κB nuclear translocation caused by NADPH oxidase-generated ROS.

MMP-2 and MMP-9 are gelatinases, belonging to a large group of proteins called extracellular matrix metalloproteinases (MMPs), and cleave elastin, type IV collagen, and several other ECM molecules [59]. Activation of MMP-2 and/or MMP-9 has been found to play an important role in the pathogenesis of diabetic retinopathy [60, 61], nephropathy [62], neuropathy [63], vascular complications [64], skin complications [65], etc. In this study, MMP-9 expression and enzymatic activity were found to be increased upon PM_2.5_ exposure in mouse alveolar macrophages and this effect was enhanced in cells with high glucose pretreatment. PM_2.5_ exposure also caused enhanced IL-1β production in alveolar macrophages with high glucose pretreatment, but there was a delay in MMP-9 upregulation as compared to IL-1β. This raised the intriguing possibility that IL-1β might regulate MMP-9 production in alveolar macrophages. To explore this possibility, cells from NLRP3 KO and IL-1R1 KO mice were used. In NLRP3 KO mice, pro-IL-1β cannot be cleaved to form mature IL-1β [34] while IL-1R1 KO mice fail to respond to IL-1β [35]. No increased expression and enzymatic activity of MMP-9 were observed in primary alveolar macrophages obtained from both NLRP3 KO and IL-1R1 KO mice, suggesting the important role of IL-1β in the regulation of MMP-9 production in cells exposed to PM_2.5_ with and without high glucose pretreatment. Previous studies have demonstrated that IL-1β can induce MMP-9 upregulation in multiple cells including human alveolar epithelial carcinoma cells A549 [30], human fetal neurons [31], rat glomerular mesangial cells [32], mouse cochlear cells HEI-OC1 [33], etc. IL-1β was also able to upregulate MMP-2 expression in rat cardiac microvascular endothelial cells [66]. Although the precise mechanisms that how IL-1β regulates MMP-9 expression remain unclear, several possible pathways have been explored including signaling cascades leading to the activation of AP-1 and NF-κB [32], c-Src-dependent transactivation of EGFR/PDGFR/PI3K/Akt linking to the NF-κB pathway [30], activation of ERKs and p38 MAPK signaling pathways [32, 33], etc.

## Conclusions

Pretreatment of mouse alveolar macrophages with high glucose enhanced PM_2.5_-induced production of pro-inflammatory cytokine IL-1β through activation of the NLRP3 inflammasome and increased nuclear translocation of transcription factor NF-κB due to PM_2.5_-induced oxidative stress, finally leading to MMP-9 upregulation (Fig. 12). This study provides a further understanding of the potential mechanisms underlying the susceptibility of individuals with diabetes to air pollution. The pro-inflammatory cytokine IL-1β may lead to acute and chronic pulmonary inflammation and contribute to other PM_2.5_-related health problems. Thus, the steps in the IL-1β synthesis and secretion pathway may act as potential therapeutic targets. Development of new drugs targeting the NLRP3 inflammasome, IL-1 receptor antagonists, agents that can remove IL-1β from the circulation, etc., may reduce the susceptibility of diabetics to air pollution. Antidiabetic medications that also inhibit IL-1β secretion may offer considerable therapeutic promise in regions with high PM pollution. For example, pioglitazone can reduce IL-1β secretion [67] and glyburide can inhibit the activation of the NLRP3 inflammasome [68]. In addition, to reduce PM-induced oxidative stress, a daily supplement of antioxidants such as vitamins C and E as well as fresh vegetables and fruits may also be recommended for individuals with diabetes. These findings may also inform PM regulations for susceptible populations, such as individuals with diabetes. To further confirm the susceptibility of individuals with diabetes to air pollution and the underlying mechanisms, in vivo studies involving obese or diabetic animal models may be performed.

### Supplementary Information

**Additional file 1**: Cytotoxicity of PM_2.5_ on MH-S cells. The cells were treated with PM_2.5_ for 24 h. The cells treated with physiological saline were used as control. Cytotoxicity was determined by alamarBlue^TM^ assay (Invitrogen). Data are shown as mean ± SEM (n = 6). * *p* < 0.05 vs. control.

**Additional file 2**: Enhanced expression of IL-1β and NLRP3 in primary mouse alveolar macrophages exposed to PM_2.5_ at high glucose setting. Primary alveolar macrophages were obtained from C57BL/6J mice by bronchoalveolar lavage (BAL) and pretreated with 30 mM of glucose for 24 h, followed by treatment with 50 μg/mL of PM_2.5_ for 3 h. The cells treated with physiological saline were used as control. The mRNA expressions of IL-1β and NLRP3 were determined by RT-qPCR and normalized to the β-actin expression in the same sample. Data are shown as mean ± SEM of three independent experiments. * *p* < 0.05 vs. control; # *p* < 0.05 vs. group with PM_2.5_ treatment only.

**Additional file 3**: Mitochondrial superoxide was not increased in MH-S cells exposed to PM_2.5_. The cells were pretreated with 5 µM of MitoSOX^TM^ for 1 h, followed by treatment with PM_2.5_ for 12 h. The cells treated with physiological saline were used as control. Data are shown as mean ± SEM (n = 3).

**Additional file 4**: The expression of TIMP-1 and TIMP-2 in MH-S cells exposed to PM_2.5_. The cells were treated with 25 or 50 µg/mL of PM_2.5_ for 3, 6, 12, and 24 h. The cells treated with physiological saline were used as control. The mRNA expression of TIMP-1 or TIMP-2 was determined by RT-qPCR and normalized to the β-actin expression in the same sample. Data are shown as mean ± SEM (n = 3).

**Additional file 5**: Uncropped versions of Western blots.

## Data Availability

All data and materials are included in the manuscript.

## Abbreviations

cDNA: Complementary DNA
DM: Diabetes mellitus
DPI: Diphenyleneiodonium chloride
H2DCF-DA: 2′, 7′-Dichlorodihydrofluorescein diacetate
IL-1β: Interleukin 1 beta
IL-1R1: Interleukin 1 receptor, type I
KO: Knock out
MMP-2: Matrix metalloproteinase-2
MMP-9: Matrix metalloproteinase-9
NF-κB: Nuclear factor-kappa B
NLRP3: NOD-, LRR- and pyrin domain-containing protein 3
PM_2.5_: Ambient fine particulate matter (diameter less than 2.5 µM)
PVDF: Polyvinylidene difluoride
ROS: Reactive oxygen species
SOD: Superoxide dismutase
TIMP-1: Tissue inhibitor of metalloproteinases 1
TIMP-2: Tissue inhibitor of metalloproteinases 2
TLRs: Toll-like receptors
