# Supplementary figures and images for: High glucose enhances the activation of NLRP3 inflammasome by ambient fine particulate matter in alveolar macrophages
Source: Part Fibre Toxicol. 2023 Nov 2;20:41. doi: 10.1186/s12989-023-00552-8 (PMC10621103; doi:10.1186/s12989-023-00552-8)

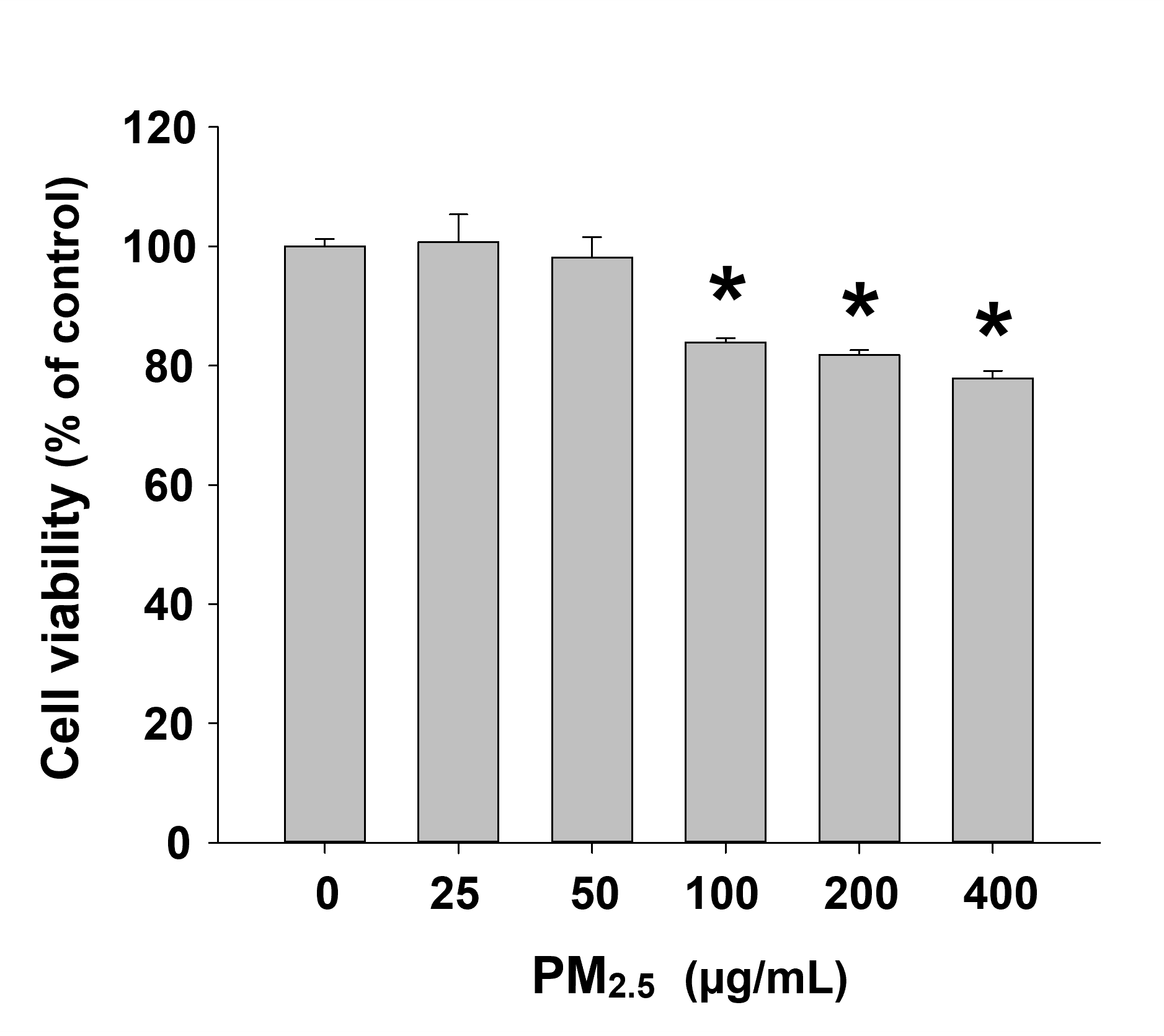

Supplement: Supplementary file 1 — Additional file 1: Cytotoxicity of PM2.5 on MH-S cells. The cells were treated with PM2.5 for 24 h. The cells treated with physiological saline were used as control. Cytotoxicity was determined by alamarBlueTM assay (Invitrogen). Data are shown as mean ± SEM (n = 6). * p < 0.05 vs. control. [file 12989_2023_552_MOESM1_ESM.tif]

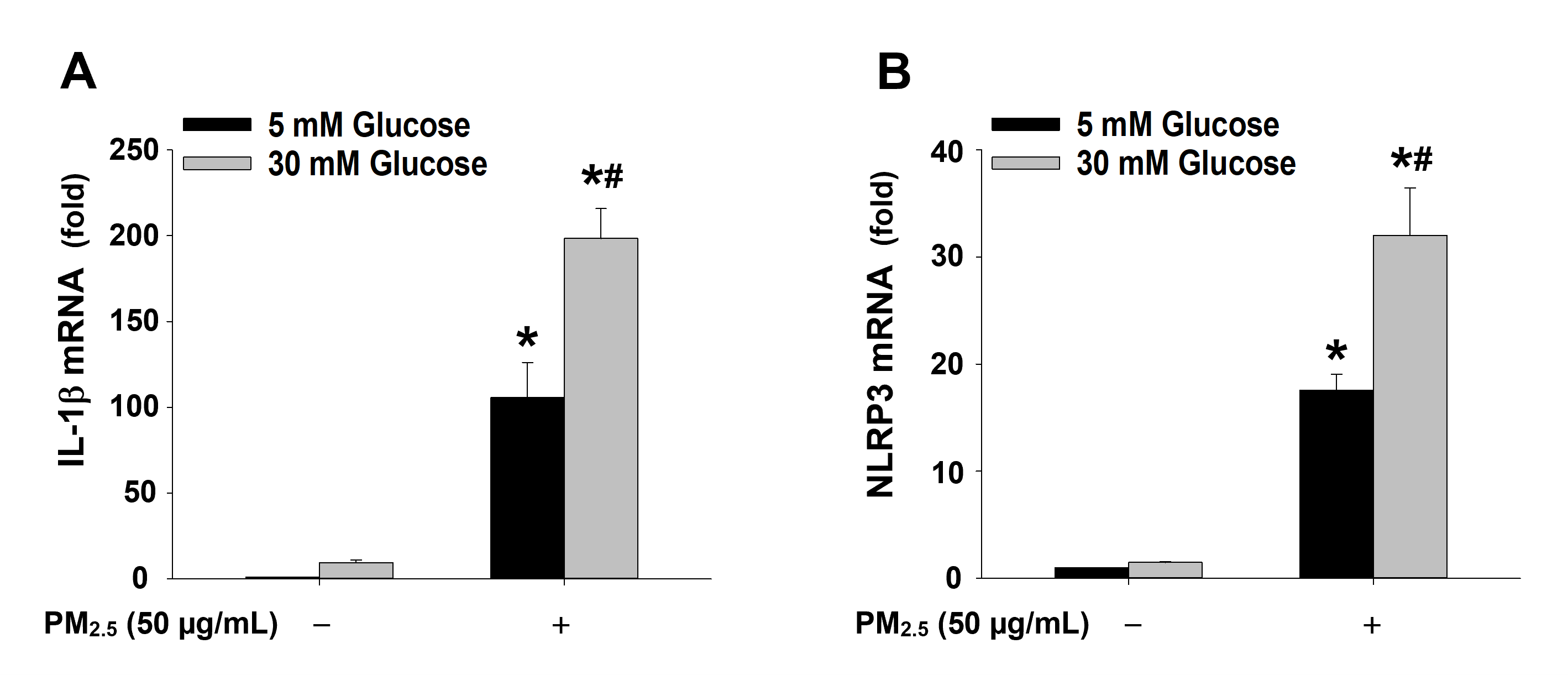

Supplement: Supplementary file 2 — Additional file 2: Enhanced expression of IL-1β and NLRP3 in primary mouse alveolar macrophages exposed to PM2.5 at high glucose setting. Primary alveolar macrophages were obtained from C57BL/6J mice by bronchoalveolar lavage (BAL) and pretreated with 30 mM of glucose for 24 h, followed by treatment with 50 μg/mL of PM2.5 for 3 h. The cells treated with physiological saline were used as control. The mRNA expressions of IL-1β and NLRP3 were determined by RT-qPCR and normalized to the β-actin expression in the same sample. Data are shown as mean ± SEM of three independent experiments. * p < 0.05 vs. control; # p < 0.05 vs. group with PM2.5 treatment only. [file 12989_2023_552_MOESM2_ESM.tif]

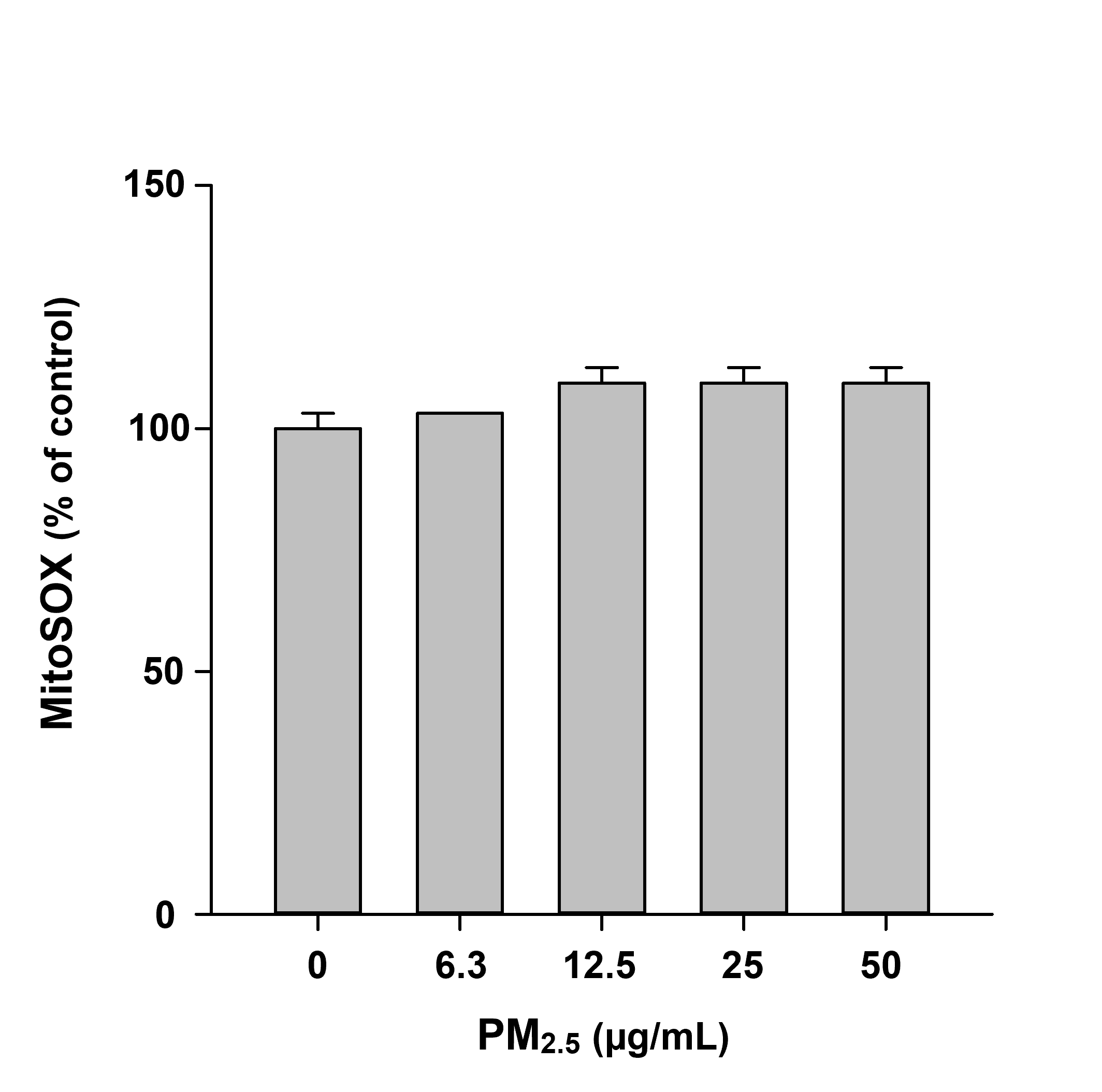

Supplement: Supplementary file 3 — Additional file 3: Mitochondrial superoxide was not increased in MH-S cells exposed to PM2.5. The cells were pretreated with 5 µM of MitoSOXTM for 1 h, followed by treatment with PM2.5 for 12 h. The cells treated with physiological saline were used as control. Data are shown as mean ± SEM (n = 3). [file 12989_2023_552_MOESM3_ESM.tif]

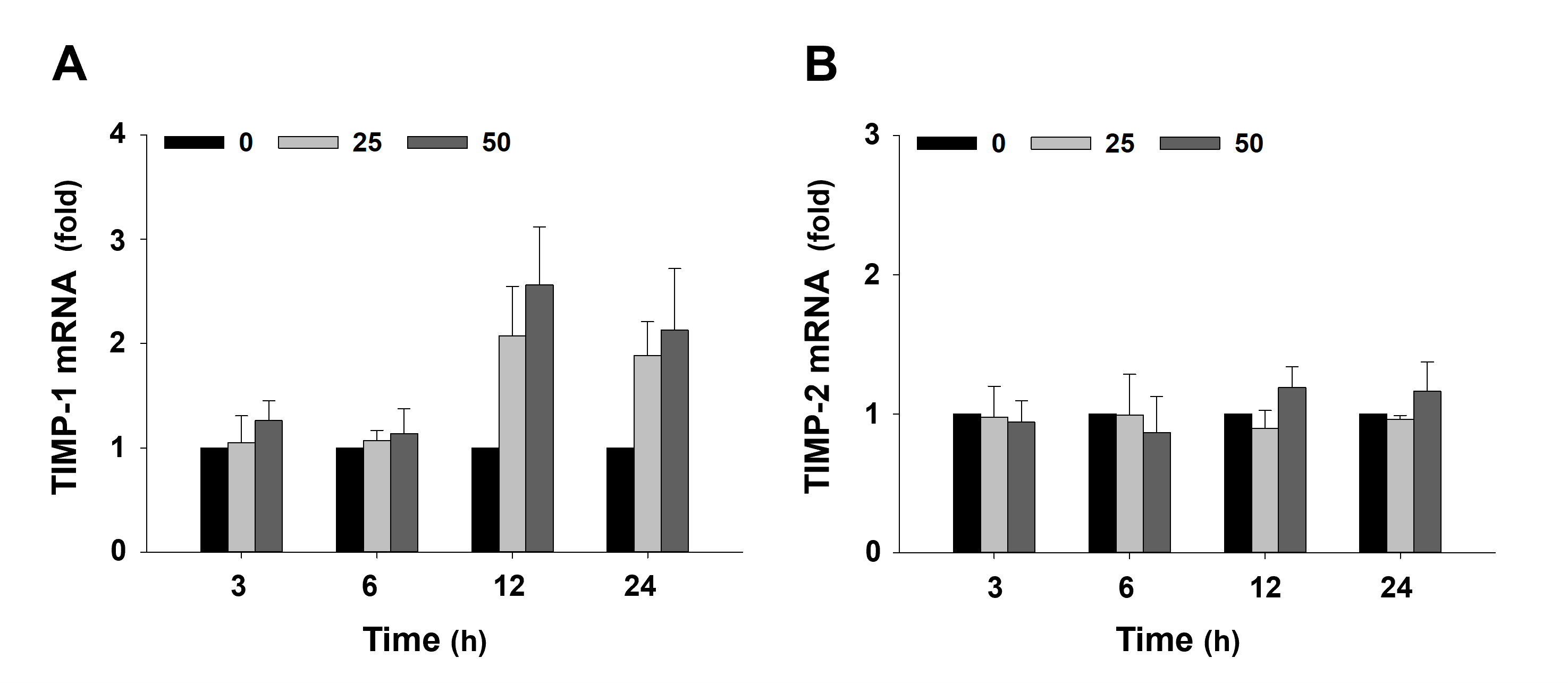

Supplement: Supplementary file 4 — Additional file 4: The expression of TIMP-1 and TIMP-2 in MH-S cells exposed to PM2.5. The cells were treated with 25 or 50 µg/mL of PM2.5 for 3, 6, 12, and 24 h. The cells treated with physiological saline were used as control. The mRNA expression of TIMP-1 or TIMP-2 was determined by RT-qPCR and normalized to the β-actin expression in the same sample. Data are shown as mean ± SEM (n = 3). [file 12989_2023_552_MOESM4_ESM.tif]
