# Supplementary material for: High glucose enhances the activation of NLRP3 inflammasome by ambient fine particulate matter in alveolar macrophages
Source: Part Fibre Toxicol. 2023 Nov 2;20:41. doi: 10.1186/s12989-023-00552-8 (PMC10621103; doi:10.1186/s12989-023-00552-8)
Supplement: Supplementary file 5 — Additional file 5: Uncropped versions of Western blots. [file 12989_2023_552_MOESM5_ESM.pdf]

Figure 3A

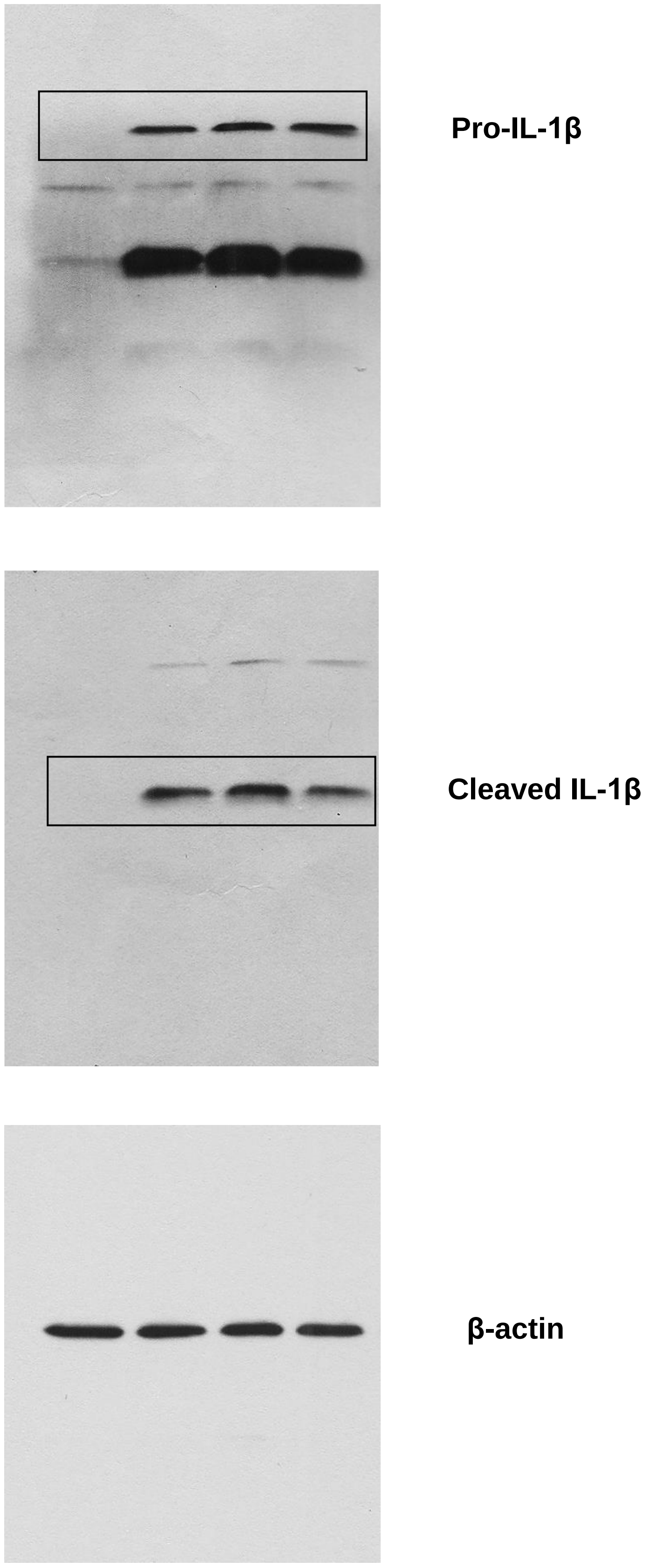

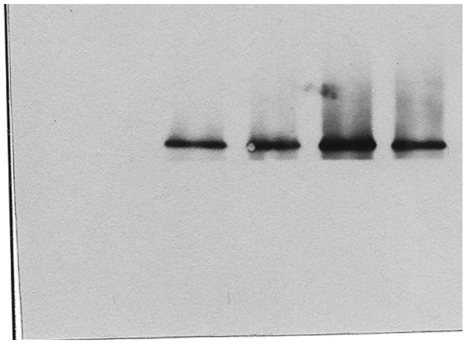

NLRP3

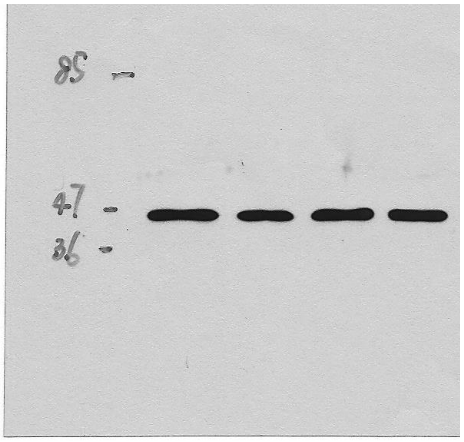

β-actin

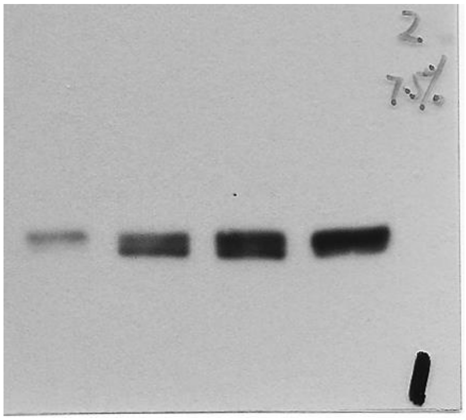

TLR2

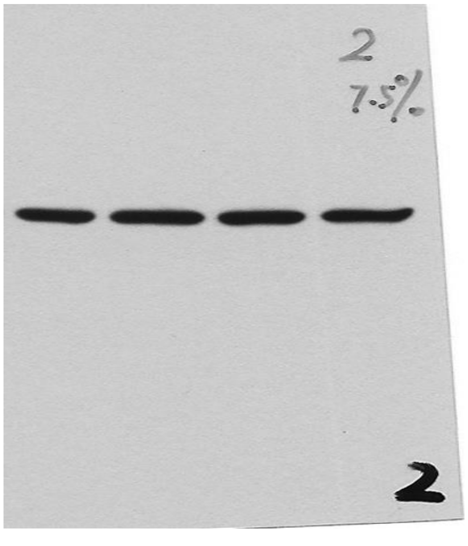

β-actin

Figure 4A

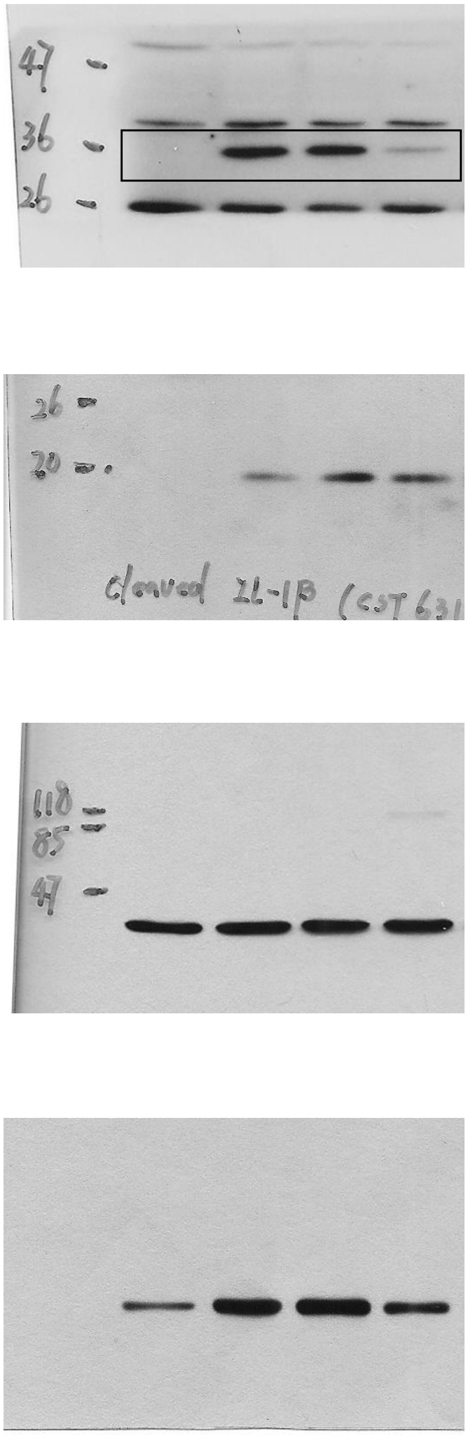

Pro-IL-1β

Cleaved IL-1β

β-actin

NLRP3

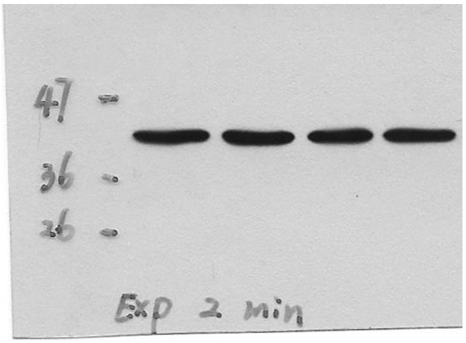

β-actin

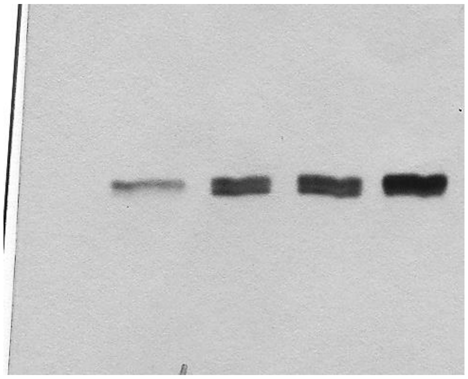

TLR2

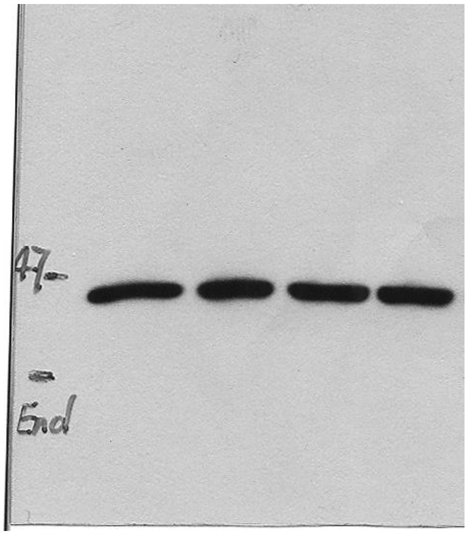

β-actin

Figure 6A

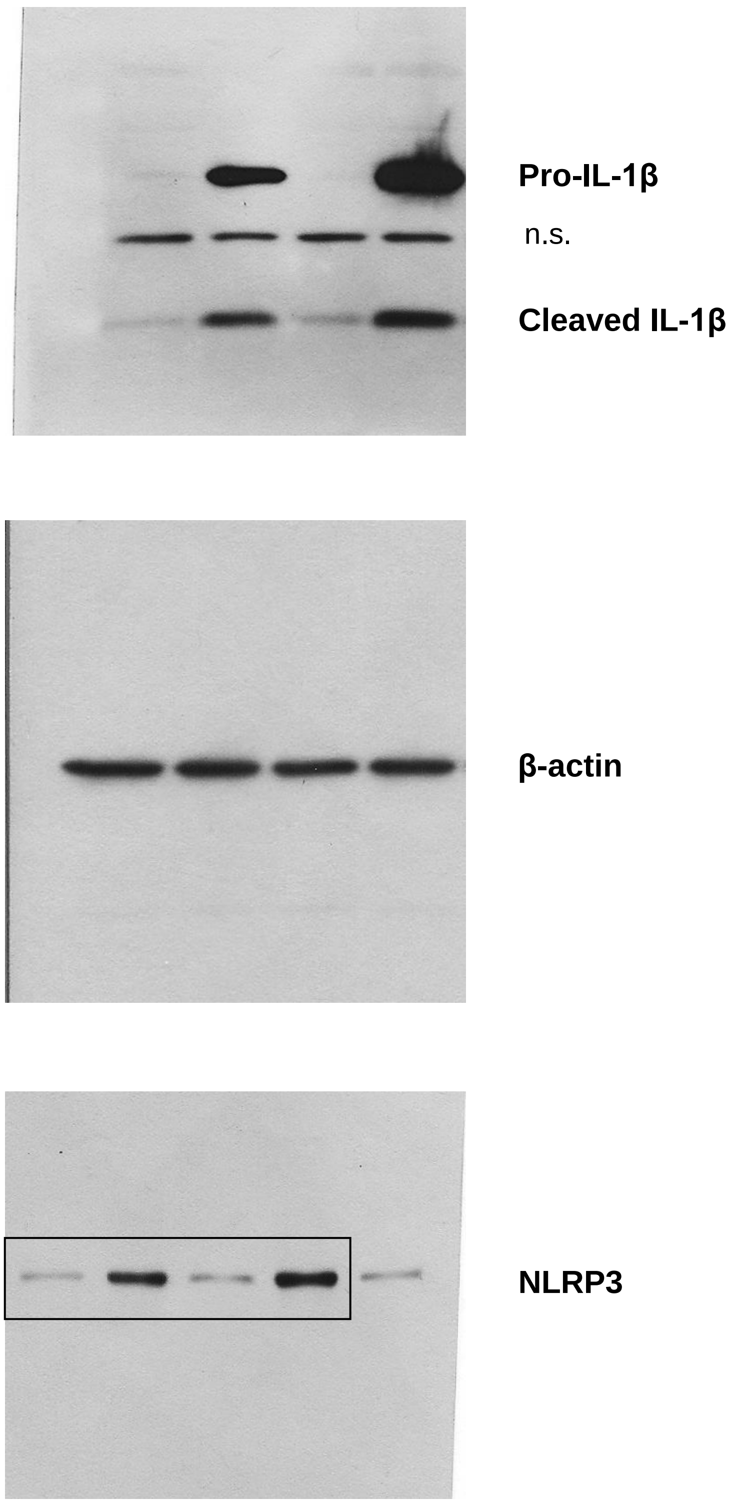

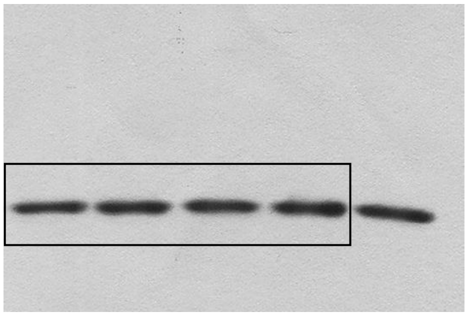

β-actin

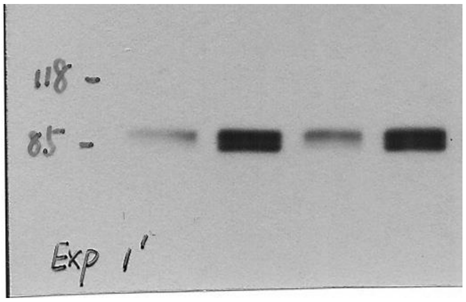

TLR2

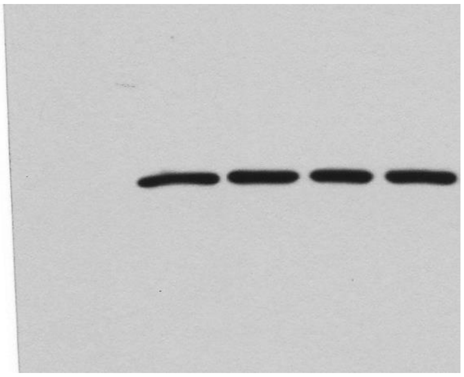

β-actin

Figure 7A

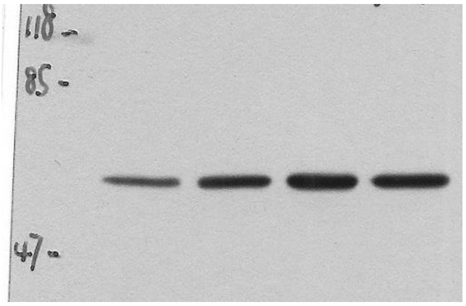

NF-κB p65 (Nuc)

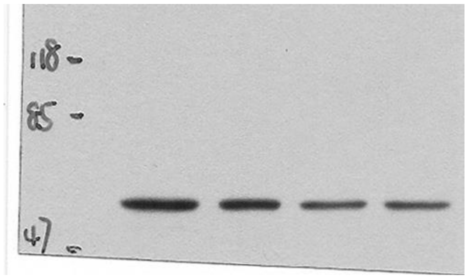

NF-κB p65 (Cyt)

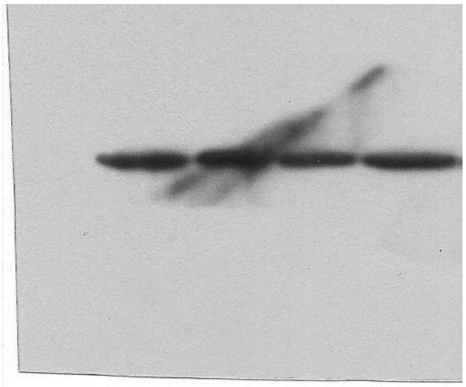

Histone H3

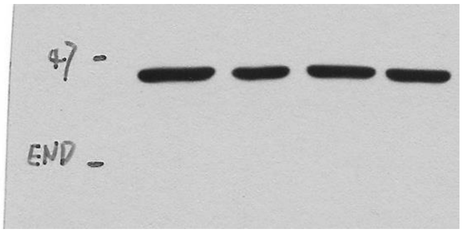

β-actin

Figure 7C

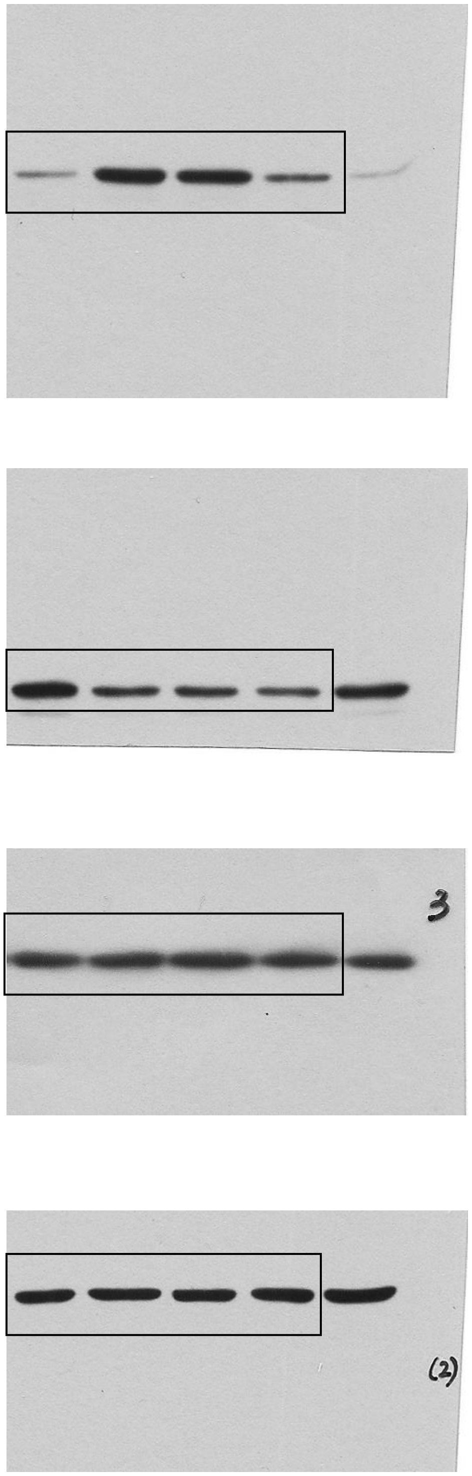

NF-κB p65 (Nuc)

NF-κB p65 (Cyt)

Histone H3

β-actin

Figure 7E

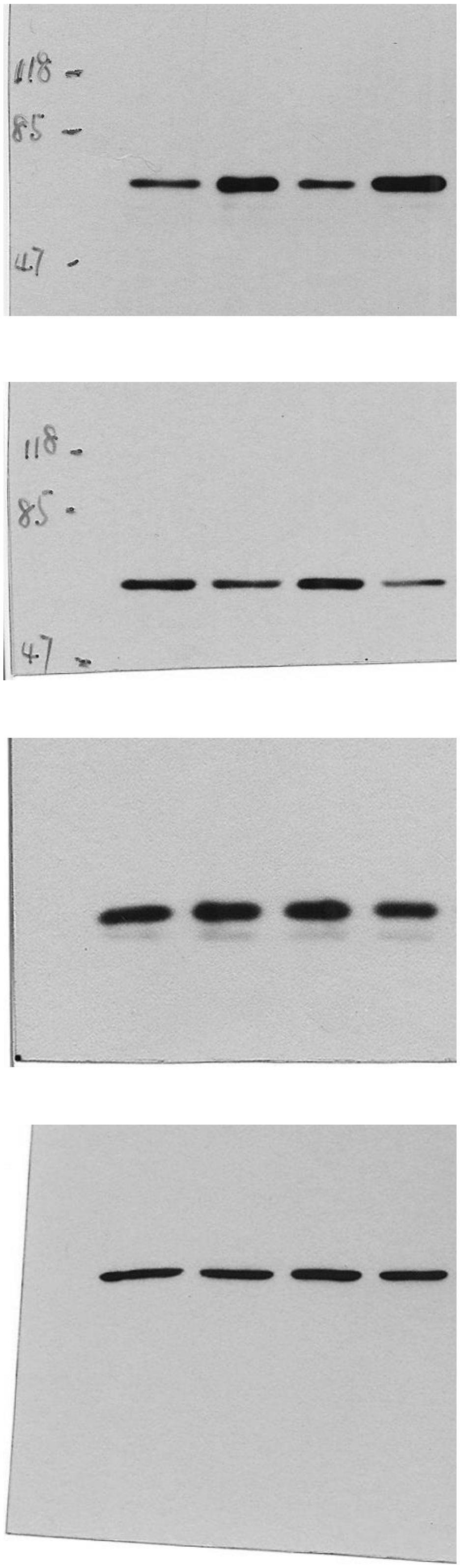

NF-κB p65 (Nuc)

NF-κB p65 (Cyt)

Histone H3

β-actin

Figure 9A

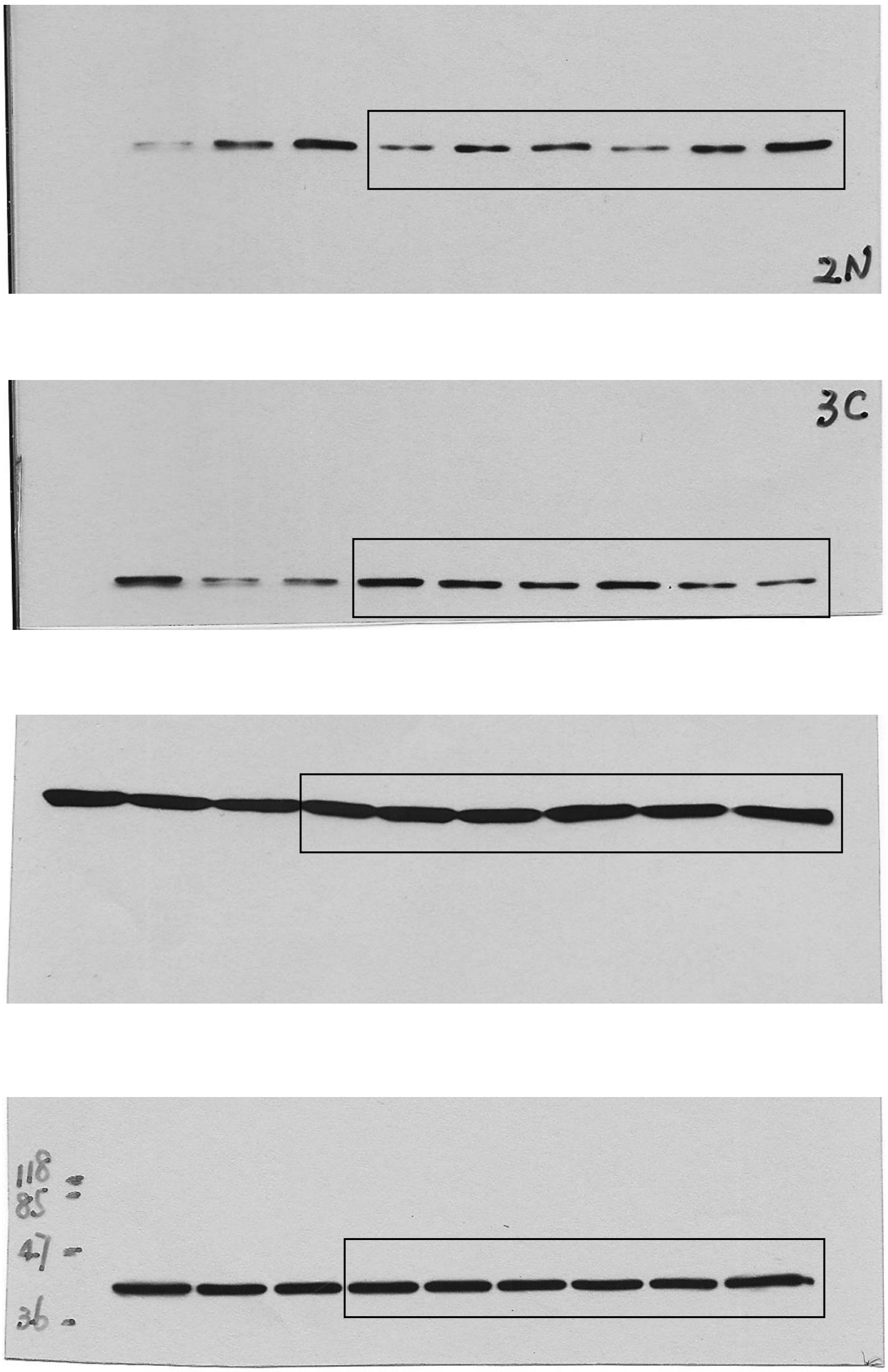

NF-κB p65 (Nuc)

NF-κB p65 (Cyt)

Histone H3

β-actin

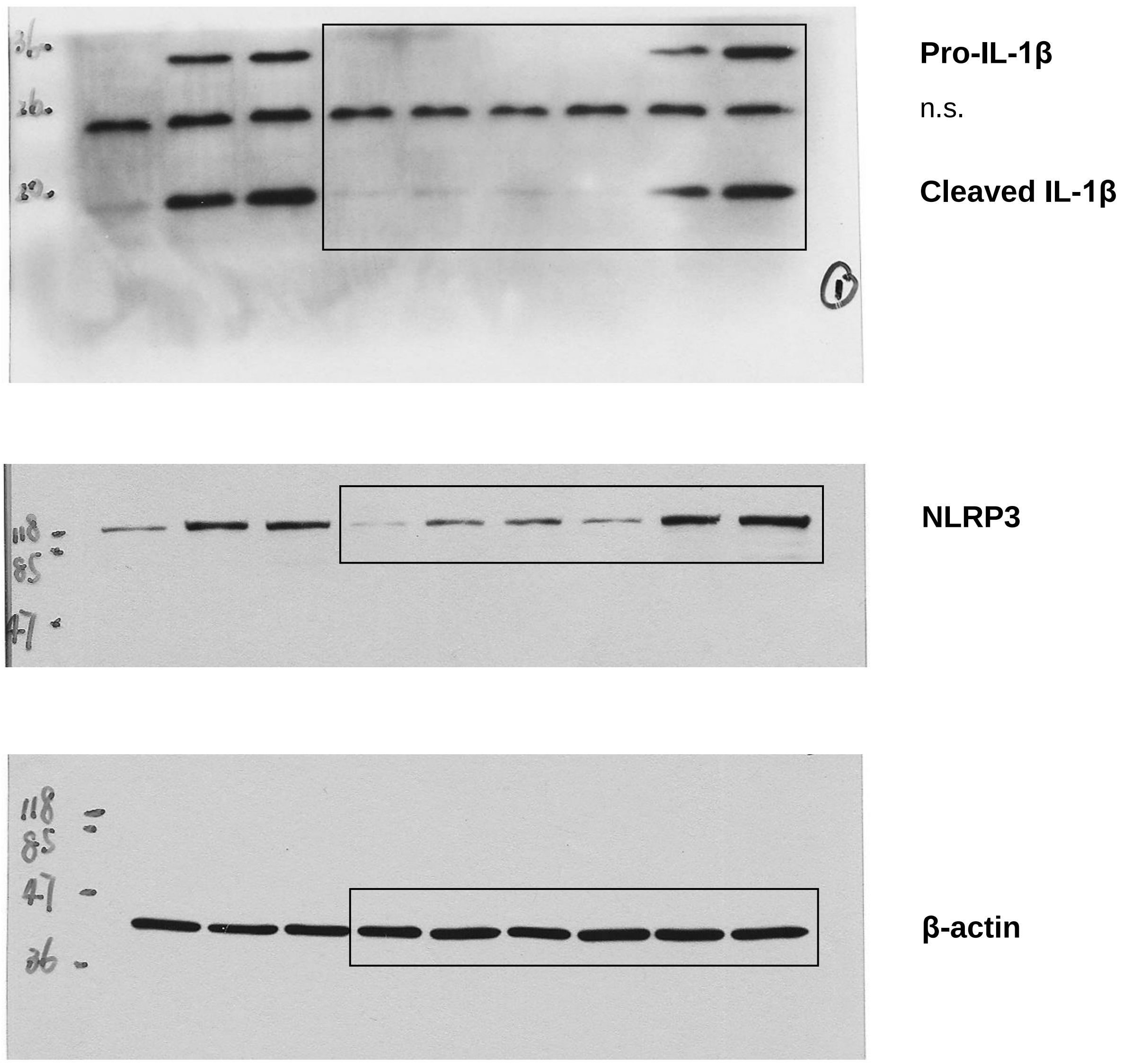
Pro-IL-1β
n.s.
Cleaved IL-1β
NLRP3
β-actin
